# Effects of chlorination on the survival of sewage bacteria in seawater microcosms

**DOI:** 10.1111/1758-2229.13216

**Published:** 2023-11-21

**Authors:** Mandy Lok Yi Tang, Stanley Chun Kwan Lau

**Affiliations:** ^1^ Department of Ocean Science Hong Kong University of Science and Technology Hong Kong China; ^2^ Center for Ocean Research in Hong Kong and Macau Hong Kong University of Science and Technology Hong Kong China

## Abstract

Chlorination is a commonly used disinfection method in sewage treatment process. However, resistant bacteria may survive chlorination and enter the receiving aquatic environment upon effluent discharge. There has been limited research on the effects of chlorination on bacterial survival in seawater. To address this knowledge gap, microcosm experiments were conducted to simulate the discharge of chlorinated effluents into coastal seawater. The results revealed that bacterial communities in seawater‐based effluents survived better in seawater than those in freshwater‐based effluents. High chlorine dosages could significantly reduce the viable bacterial populations and their chance of regrowth in seawater. Additionally, faecal indicator bacteria (FIB) that entered the viable but non‐culturable (VBNC) state under chlorination tended to persist in the VBNC state without resuscitation during seawater incubation. Because of the prevalence of VBNC indicator bacteria, qPCR quantification of FIB was more effective than conventional culture‐based methods in tracing viable pathogenic chlorine‐resistant bacteria, although the correlation strength varied depending on the type of effluent. This study sheds light on how chlorine dosages and the intrinsic properties of effluents affect bacterial survival in seawater and highlights the potential and limitations of using FIB in monitoring the health risks associated with the discharge of chlorinated effluents.

## Graphical abstract

Bacteria from saline‐based effluents survived better in seawater than those from freshwater‐based effluents. High chlorine dosages reduced the chance of bacterial regrowth in seawater. Due to the prevalence of VBNC cells in chlorinated effluents, qPCR quantifications of faecal indicator bacteria were more effective than culture‐based methods in tracing chlorine‐resistant bacteria.

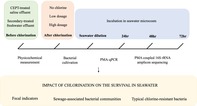

## INTRODUCTION

The discharge of improperly treated effluents introduces excess nutrients, pathogens, heavy metals, antibiotics and other pharmaceutical products to coastal seawater (Su et al., 2022; Wear et al., 2021), leading to problems such as algal blooms, hypoxia, aquatic toxicity, antibiotic resistance and waterborne diseases. These issues seriously compromise the ecosystem and public health. Although sewage treatment facilities employ disinfection to treat effluents before discharge, some bacteria may persist and be introduced into the receiving environment. The incomplete removal of bacteria after disinfection may arise from practical considerations, such as the cost of disinfection processes, formation of disinfection byproducts and types of effluent reuse. Additionally, unintended consequences of microbial disinfectant resistance, biofilm formation on distribution pipes, outdated discharge standards and monitoring schemes may also contribute to the persistence of bacteria (Chandy & Angles, 2001; Naidoo & Olaniran, 2013; Tang & Lau, 2022; Xie et al., 2022). Therefore, designing an effective disinfection scheme requires careful consideration of the intended outcomes and in‐depth understanding of microbial responses.

Bacteria can utilise various mechanisms to protect themselves against disinfection. One of the strategies is altering their physiological states. Common disinfection methods such as chlorination can significantly reduce the number of culturable bacterial cells. However, some bacterial cells can enter the viable but non‐culturable (VBNC) state in response to chlorine‐induced sublethal stress. The VBNC state allows them to maintain membrane integrity and metabolic activities despite the loss of culturability (Li et al., 2014; Schottroff et al., 2018). Reports have shown that some VBNC bacteria can regain culturability and proliferate after the alleviation of chlorine stress (Lin et al., 2017; Oliver et al., 2005). This poses a significant public health concern as culture‐based monitoring methods commonly adopted by regulatory agencies around the world cannot detect VBNC bacteria, leading to an underestimation of the presence of viable cells after disinfection. On the other hand, some bacterial strains have particularly strong resistance against chlorine. They are regarded as chlorine‐resistant bacteria (CRB). Their resistance depends on cellular permeability barriers and chlorine consuming substances. Cellular structures such as cell membranes, cell walls and extracellular polymeric substances (EPS) provide diffusion barriers, while organic substances such as fatty acids and amino acids contributes to the consumption of chlorine (Cloete, 2003; Furuhata et al., 2014; Wang et al., 2019). CRB are frequently found in sewage, and many of them are opportunistic pathogenic (Luo et al., 2021). Although there is no evidence indicating a direct linkage between bacterial chlorine resistance and pathogenicity, it was reported that reactive chlorine species could enhance the expression of genes required for bacterial virulence (Nontaleerak et al., 2020). Since CRB can survive and reproduce under sublethal chlorine dosages while other bacteria are susceptible to chlorine oxidative stress, chlorination can lead to an enrichment of pathogenic CRB in the effluents.

Bacteria that survive sewage treatment and disinfection processes will be exposed to the receiving environment upon discharge. Although faecal bacteria originally inhabit animal intestines, some may persist in the external environment for a long period (Solecki et al., 2011; Zhang et al., 2015). Regrowth of bacteria was sometimes observed (Desmarais et al., 2002). Evaluating the fate of sewage‐associated bacteria in the receiving coastal water and the resilience of the ecosystem to effluent disturbance are vital for water quality management and remediation. While studies have been focusing on the impact of environmental factors such as sunlight, salinity, temperature and organic matter on the survival of sewage‐associated bacteria (Ahmed et al., 2018; Korajkic et al., 2013; Rochelle‐Newall et al., 2015), there is limited research on the relationship between chlorination and bacterial survival after releasing into the environment. Chlorine dosage determines not only the magnitude of bacterial removal during disinfection but also the types of bacteria that remain in the disinfected effluent and subsequently enter the receiving water. Unlike the highly variable environmental factors, chlorine dosage can be controlled during the sewage treatment processes. Therefore, evaluating the impact of chlorine dosage on the fate of sewage‐associated bacteria in the receiving water can offer valuable insights for sustainable management of water resources.

In this study, indoor microcosm experiments were designed to simulate the discharge of chlorinated effluents into coastal seawater. Seawater‐based primarily treated and freshwater‐based secondarily treated effluents were collected from two municipal sewage treatment works. In the lab, the effluents were treated with different chlorine dosages. After dechlorination, the effluents were diluted with seawater, sealed in dialysis bags and incubated in microcosms with flow‐through coastal seawater for 3 days. The settings of microcosms simulated the conditions of effluent discharge from submarine outfalls, including effluent‐seawater dilution factors and benthic water conditions. Bacterial cultivation, quantitative polymerase chain reaction (qPCR) coupled with propidium monoazide (PMA) treatment, 16S rRNA amplicon sequencing and SourceTracker analysis were used to investigate the physiological states (i.e., total, viable and culturable), chlorine resistance and survival of two common faecal indicator bacteria (FIB) (*Escherichia coli* and *Enterococcus*), typical CRB, sewage‐associated bacterial communities and indigenous seawater bacterial communities.

The study aimed to achieve three main objectives: (i) determine the effects of chlorine dosages on the total, viable and culturable populations of FIB after chlorination and discharge into the seawater, (ii) track the population dynamics of the total and viable effluent and seawater bacterial communities after effluent discharge and (iii) assess the performance of FIB in predicting the potential bacteriological threats associated with CRB in chlorinated effluents.

## EXPERIMENTAL PROCEDURES

### Collection of treated effluents

Primarily and secondarily treated effluents before disinfection were collected from two municipal sewage treatment works in Hong Kong. Primarily treated effluents were collected from Stonecutters Island Sewage Treatment Works (SC), which is one of the largest primary sewage treatment plants in the world, with a treatment capacity of 1.9 × 10^6^ m^3^ d^−1^. It receives saline influent from urban areas using seawater for toilet flushing and applies chemically enhanced primary treatment (CEPT). Ferric chloride and polymer are mixed with the sewage inflow during CEPT. After flocculation and sedimentation, the sludge and scum are dewatered and removed.

In contrast, secondarily treated effluents were collected from Stanley Sewage Treatment Works (ST), which is a relatively small‐scale secondary treatment facility (8.8 × 10^3^ m^3^ d^−1^) for treating influent collected from areas using freshwater for toilet flushing. In ST, the sewage undergoes screening and degritting to remove coarse suspended solids before flowing through aeration tanks for secondary treatment, with a retention time of around 15 h. After that, the activated sludge and scum are separated from the treated effluent in sedimentation tanks. Both SC and ST disinfect treated effluents by adding sodium hypochlorite (NaOCl) solution before disposal to coastal seawater via submarine outfall.

In this study, 50 L of treated effluents were collected from each treatment work before the chlorination. The effluents were transported to the lab on ice in autoclaved polypropylene (PP) bottles. Triplicates of 1 L treated effluents from each treatment work were set aside for sample analyses (as described in Sections Measurement of physicochemical parameters, Statistics analysis and data visualisation), while the remaining effluents were used for chlorine treatment.

### Chlorination

The SC and ST effluents were each divided into three disinfection treatments (high chlorine dosage, low chlorine dosage and without chlorination). The high dosage treatment aimed to achieve around 4 log_10_ reduction in the culturable plate count of *E. coli*, while the low dosage aimed for around 2 log_10_ reduction. These disinfection targets were set according to the upper and lower ranges of the monthly disinfection outcome in SC (https://www.dsd.gov.hk). Based on trial experiments, 9.0 and 7.0 mg L^−1^ of NaOCl were used for the high and low dosage treatments of SC effluents, respectively. For ST effluents, 2.0 and 1.2 mg L^−1^ of NaOCl were used for the high and low dosage treatments, respectively. Effluents without chlorination were added with the same volume of autoclaved phosphate‐buffered saline (PBS) in lieu of NaOCl.

Each treatment was performed in triplicates of 5 L effluent. NaOCl or PBS was added to the effluents in glass beaker with continuous mixing by magnetic stirrers. The contact time of the treatment was 20 min, in accordance with the time for chlorination in SC and ST. At the end of the treatment, effluents treated with NaOCl were sampled for the measurement of total residual chlorine (TRC), followed by the addition of 0.1 M sodium thiosulphate in excess to terminate the chlorination. TRC was measured in triplicates using the DPD chlorine colorimeter (YSI, Inc.) according to the manufacturer's instruction. The TRC data are provided in Table S1. Triplicates of 1.5 L effluents from each treatment were set aside for sample analyses (as described in sections Measurement of physicochemical parameters, Statistics analysis and data visualisation), while the remaining effluents were used for the microcosm experiment.

### Microcosm experiment

After disinfection treatments, effluents were diluted 1:50 with freshly collected seawater. The dilution ratio was chosen based on the approximate median level of effluent dilution within the initial mixing zone (around 500 m radius) of the submarine outfall of SC (Choi et al., 2009). The effluent‐seawater mixtures were transferred into dialysis bags and incubated in seawater microcosms for 3 days. Each microcosm consisted of a 147 × 71 × 23 cm (L × W × H) tank with continuous flow‐through of seawater. Four sets of microcosms were used to incubate the effluents from each sewage treatment work, three for the disinfection treatments (i.e., high dosage, low dosage and no chlorine) and one for seawater control (i.e., seawater without effluent mixing).

The seawater used for dilution and incubation was pumped into the lab from the coastal bottom layer at the pier of the Hong Kong University of Science and Technology through pipelines. The seawater was filtered through 1 μm membrane to remove suspended solids before use. The water temperature and salinity of the microcosms for SC samples were set to be 25.4°C and 31.9 PSU, respectively, while those for the ST samples were 23.7°C and 34.5 PSU, respectively, determined by measuring the bottom water layers at actual effluent discharge points using Conductivity, Temperature, Depth (CTD) Sensors. The microcosms were covered with curtains throughout the incubation period to mimic the dim submarine environment at the discharge points.

The dialysis bags were made of 500 mm (length) × 77‐mm (diameter) regenerated cellulose dialysis membranes sealed with sterilised plastic bag clips. The pore sizes had a molecular weight cutoff of 14 kDa to allow high rates of molecular exchange (Korajkic et al., 2014). The dialysis bags were rinsed and stored in autoclaved Milli‐Q water at 4°C before use. During the microcosm experiments, the dialysis bags were submerged below the water surface by anchoring porous plastic mats over them. Triplicate dialysis bags each containing 1.5 L of effluent‐seawater mixture or seawater were retrieved from each microcosm at four time points: immediately after the mixing of effluent and seawater (T0), 24, 48 and 72 h (T1–T3). The samples retrieved from the microcosms, together with the samples set aside before and after chlorine disinfection (described in Sections 2.1 and 2.2), accounted for a total of 120 samples for downstream analysis (Sections Measurement of physicochemical parameters, Statistics analysis and data visualisation).

### Measurement of physicochemical parameters

The temperature, salinity, dissolved oxygen (DO), pH and turbidity of the samples were measured using a multiparameter water quality sonde (YSI, USA). Meanwhile, biological oxygen demand (BOD_5_), total nitrogen (TN), ammonia (NH_3_), nitrite (NO_2_^−^), nitrate (NO_3_^−^), total phosphorous (TP), phosphate (PO_4_^3−^) and silicate (SiO_3_^2−^) were measured according to the APHA methods (APHA, 2005). Each sample were measured in triplicates and averaged. The results of the physicochemical parameters measurements were summarised in Table S1.

### *Enumeration of culturable* E. coli *and* Enterococcus *spp.*

The concentrations of culturable *E. coli* and *Enterococcus* spp. in each sample were determined using the membrane filtration method (USEPA, 2000). The samples were serially diluted using PBS (up to 10^7^‐fold) and filtered through 47 mm diameter, 0.45 μm pore‐sized cellulose nitrate membranes in triplicates. For the enumeration of *E. coli*, the membranes were incubated in dark for 24 h at 37°C on CHROMagar™ ECC agar (Ho & Tam, 1997). For *Enterococcus*, the incubation was 24 h at 41°C and mEI agar was used (USEPA, 2000). Blue colonies were counted after incubation. The concentrations of *E. coli* and *Enterococcus* were reported as numbers of colony forming units (CFU) per 100 mL of sample.

### PMA treatment, DNA extraction and qPCR

To quantify the viable populations of total bacteria, *Enterococcus* and *E. coli*, the samples were treated with PMA before DNA extraction, following the procedures in our previous study (Tang & Lau, 2022). Briefly, triplicates of 500‐mL aliquots from each sample were centrifuged and resuspended in autoclaved PBS, then added with a final concentration of 100 μM of PMA. Another set of triplicates was added with PBS in lieu of PMA for the quantification of total populations. The triplicates with or without PMA were all spiked with 1 μL of salmon testes DNA (Sketa DNA) (Sigma‐Aldrich) to evaluate PMA treatment efficiency. After dark incubation and photoactivation, each set of triplicates was pooled for DNA extraction using AllPrep® DNA/RNA Mini Kit (Qiagen). 1 μL of TaqMan® Universal DNA Spike‐In Control plasmid (Thermo Fisher Scientific, Inc.) was added to each sample after the cell lysis step as a control for DNA loss during the extraction and purification steps.

The extracted DNA was then tested with TaqMan probe‐based qPCR assays to quantify the copy numbers of the gene markers of total bacteria, *Enterococcus*, *E. coli*, salmon DNA (PMA control) and the Universal DNA Spike‐In Control (extraction control). The primer and probe sets used for the bacterial and salmon DNA gene targets were the same as those in the previous study, while that of the Universal DNA Spike‐In Control followed the proprietary TaqMan Assay (Assay ID Ac00010014_a1, Thermo Fisher Scientific Inc.) (Table S2). The reaction mixture was prepared by adding 5 μL AceQ U+ Universal Probe Master Mix V2 (Vazyme Biotech), 0.2 μM each primer, 0.1 μM TaqMan probe, 1–3 μL DNA sample and topped up to 10 μL with nuclease‐free water. The thermal cycle was 95°C for 10 min, 45 cycles of 95°C for 15 s and 60°C for 1 min. Standard curves were constructed using plasmid DNA of the target genes, following the procedures in Liu's study (Liu et al., 2015).

PCR inhibition was evaluated by the differences in *C*_*t*_ values between 10‐fold dilutions of the sample. Differences of less than 3.2 (equivalent to 91.9% efficiency) were regarded as amplification inhibition, and a higher level of dilution would be needed to alleviate the inhibition. The final gene copy numbers were corrected by the DNA extraction and PMA treatment efficiencies using the recovered gene copies of the extraction and PMA controls (Tang & Lau, 2022), calculated as below:

$$\begin{array}{l} \text{Extraction efficiency}\left(\%\right)=\frac{\text{Detected TaqMan control}\;\mathrm{DNA}\;\text{copy}}{\text{Spiked TaqMan control}\;\mathrm{DNA}\;\text{copy}}\;\times 100\% \end{array}$$

$$\mathrm{PMA}\;\text{efficiency}\left(\%\right)=\begin{array}{l} \left(1-\frac{\text{Detected salmon}\;\mathrm{DNA}\;\text{copy}}{\text{Spiked salmon}\;\mathrm{DNA}\;\text{copy}}\right)\\ \times 100\% \end{array}$$

The corrected gene copies of *E. coli* and *Enterococcus* were converted to cell equivalents by dividing the median of gene copy number per cell (7 for *E. coli* and 6 for *Enterococcus*).

### Amplicon sequencing and data processing

The V3‐V4 regions of the bacterial 16S rRNA genes in extracted DNA were amplified by using the primers 341F (5′‐CCTAYGGGRBGCASCAG‐3′) and 806R (5′‐GGACTACNNGGGTATCTAAT‐3′) for amplicon sequencing. The resulting 466 bp amplicons were sequenced on Illumina platform to generate 250 bp paired‐end raw reads, which were then processed through the QIIME 2 v2022.2 pipeline (Bolyen et al., 2019) The merging, quality filtering, denoising and chimaera removal of the reads were executed by DADA2 using the default settings (Callahan et al., 2016). Taxa were classified by the classify‐sklearn method of the q2‐feature‐classifier plugin with a classifier trained on Silva v132 99% identity clustered reference database (https://www.arb-silva.de/download/archive/).

SourceTracker 2, a software that uses Bayesian model to estimate the proportions of source communities in an unknown mixture of communities (Knights et al., 2011), was used to track the changes in the proportions of effluent and seawater bacterial communities in the effluent‐seawater mixtures over the course of the microcosm experiment (https://github.com/biota/sourcetracker2). The effluents after disinfection treatments (high dosage, low dosage and no chlorine) and the seawater controls were regarded as two types of sources, while the effluent‐seawater mixtures were regarded as sinks. The genus read counts of sources and sinks input for the analysis were rarefied to 1000 to prevent samples with more counts from dominating the proportions. Genera that could not be classified as effluent or seawater sources were regarded as unknown sources.

### Statistics analysis and data visualisation

For the alpha‐ and beta‐diversity analyses, the filtered reads were rarefied to a depth of 41,169 reads. This cutoff value was determined by the least filter read among the samples with observed sequence variants reaching plateau at the current sequencing depth in alpha rarefaction curve. Five samples with number of reads fewer than the cutoff value were discarded for the diversity analyses, including SC_LD_T0_P_1, SC_HD_T0_P_1, SC_HD_T0_P_3, ST_N_T1_P_2 and ST_N_T1_P_3 (N: no chlorine, LD: low dosage, HD: high dosage; P: with PMA treatment; 1–3: replicates).

Detrended correspondence analysis (DCA) was conducted using the vegan package in R software (https://www.r-project.org/) to determine whether a linear or unimodal model should be applied in constrained ordination analysis. Constrained correspondence analysis (CCA) plot was chosen as a unimodal model in this study as the length of the first axis in DCA ordination exceeded 4 standard deviations. Permutational Multivariate Analysis of Variance (PERMANOVA) tests were performed to identify significant constraining variables in CCA.

First order decay model was applied to calculate the linear regression of concentrations of FIB (ln cell equivalent or CFU 100 mL^−1^) against time. Kruskal‐Wallis analysis and paired *t*‐test were used to test the significance of different groups of comparisons, such as the differences in diversity indices, decay rates, relative abundances and concentrations of bacteria. The chlorine‐resistant bacteria (CRB) in the amplicon sequences were selected based on the 44 typical CRB described in Luo et al.'s study (2021). The concentrations of CRB were calculated by multiplying the relative abundances of CRB (the number of annotated CRB reads over the number of total annotated bacterial reads) by the copy numbers of total bacterial 16S rRNA genes determined in qPCR (Acharya et al., 2020; Jian et al., 2020; Tettamanti Boshier et al., 2020). Spearman's rank correlation test was utilised to estimate the correlations between the concentrations of CRB and FIB.

The visualisation of taxa plot was supported by MicrobiomeAnalyst website (https://www.microbiomeanalyst.ca/). The CCA, heatmap and correlation plots were constructed by the vegan, ggplots, RColorBrewer and corrplot packages in R. The bubble plot of the relative abundances and concentrations of CRB was constructed using Prism 9 software (https://www.graphpad.com/scientific-software/prism/).

## RESULTS

### Survival of total, viable and culturable populations of FIB under different chlorine dosages and during seawater incubation

**FIGURE 1:**
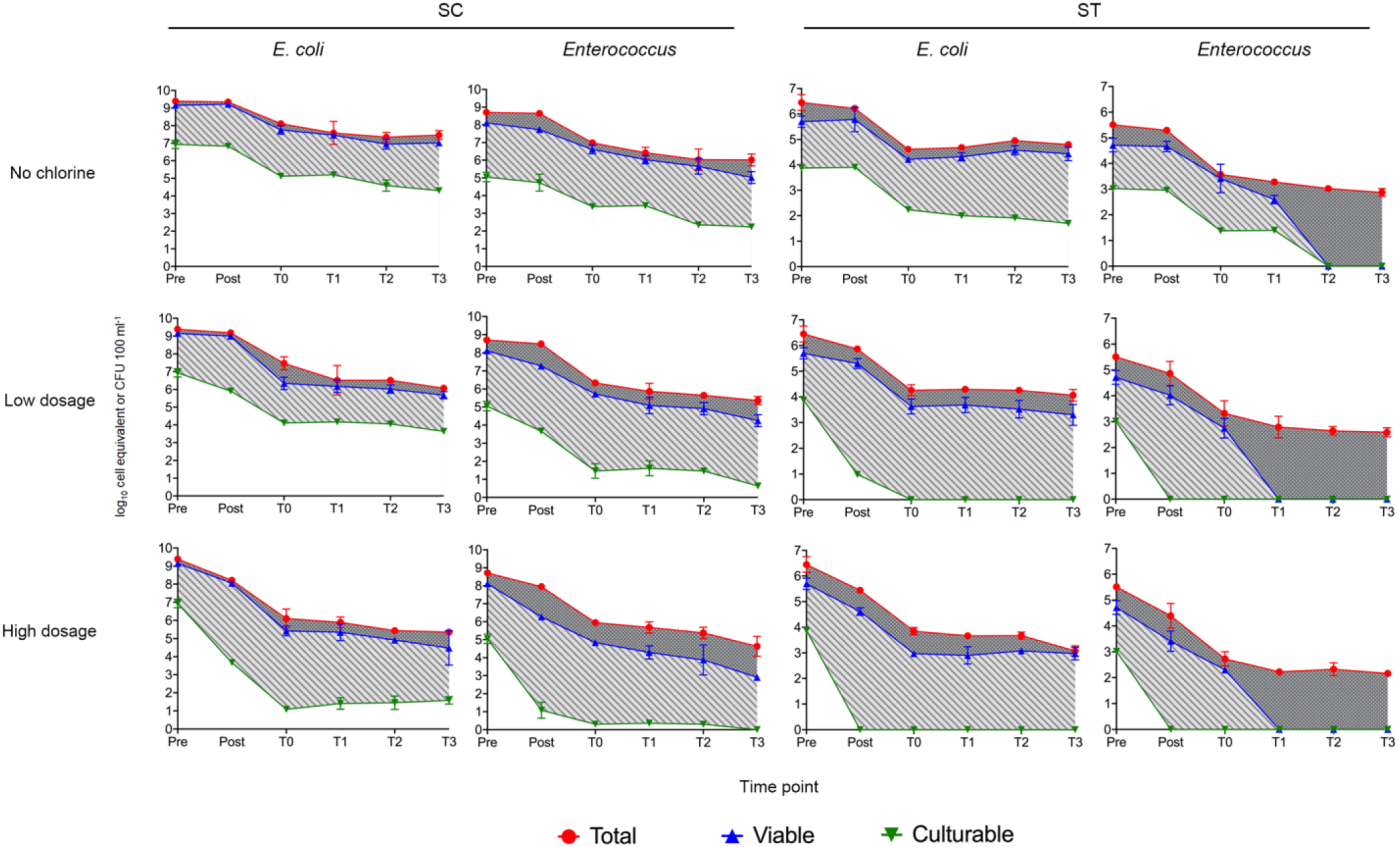
Total, viable and culturable concentrations of *Escherichia coli* and *Enterococcus* in the effluents of two sewage treatment works under different chlorine dosages. The total and viable 23S rRNA gene copy numbers detected in qPCR assays were converted to numbers of cell equivalent (log_10_ cell equivalent 100 mL^−1^) by dividing the median gene copies per cell, for comparison with the culturable plate count (log_10_ CFU 100 mL^−1^). Data are shown as mean ± 1 S.D. log_10_ copies per 100 mL of effluent derived from three replicates. Pre and post indicate samples before and after chlorination, respectively. T0 indicates samples upon dilution with seawater, T1–T3 indicate samples taken from the microcosms at 24, 48 and 72 h. Shaded area illustrates the difference between total, viable and culturable proportions.

The reduction in the culturable plate counts of *E. coli* and *Enterococcus* increased with chlorine dosages (Figure 1). The average culturable counts of *E. coli* in the SC effluents reduced by approximately 1 log_10_ CFU 100 mL^−1^ under low dosage and over 3 log_10_ CFU 100 mL^−1^ under high dosage, while those in the ST effluents reduced by over 2 log_10_ CFU 100 mL^−1^ under low dosage and became undetectable (<1 CFU 100 mL^−1^) under high dosage. For *Enterococcus*, the culturable counts in SC effluents reduced by over 1 and 4 log_10_ CFU 100 mL^−1^ under low and high dosage, respectively, while those in ST effluents were below detection under both dosage conditions. On the contrary, the culturable counts of *E. coli* and *Enterococcus* in the SC and ST effluents without chlorination differed by less than 0.3 log_10_ CFU 100 mL^−1^.

Upon 50‐fold dilution with seawater at T0, the culturable counts of *E. coli and Enterococcus* reduced by 1–2 log_10_ CFU 100 mL^−1^ in SC samples. During the 72 h of incubation, the average culturable counts of *E. coli* in the SC samples treated with low dosage reduced gradually. However, there was a slight rise in the culturable counts of *E. coli* under high dosage. Meanwhile, the culturable counts of *Enterococcus* in SC samples declined regardless of the dosage. For the ST samples, the culturable counts of *E. coli* and *Enterococcus* after chlorination were undetectable throughout the incubation period. No culturable *E. coli* and *Enterococcus* were observed in the seawater control.

For the quantification of total and viable FIB, the copy numbers of the gene markers detected in qPCR were corrected by DNA extraction and PMA treatment efficiencies. The average DNA extraction efficiency of the samples was 1.45 ± 0.48% (mean ± 1 S.D.), while the average PMA treatment efficiency was 92.85 ± 8.22%. The concentrations of total and viable *E. coli* and *Enterococcus* cells were around 2–7 log_10_ higher than their culturable cells on average (Figure 1). The differences between viable and culturable concentrations increased with chlorine dosage. During seawater incubation, the concentrations of viable *Enterococcus* decreased more rapidly than viable *E. coli*. The viable concentrations of *E. coli* changed only slightly over the incubation period regardless of the dosages. The gene markers of total and viable *E. coli* and *Enterococcus* were undetectable (<1 log_10_ gene copies μl^−1^) in the seawater control.

In the majority of experimental sets, bacterial concentrations declined throughout the seawater incubation period (Table 1), with the exception of the culturable *E. coli* cells in SC samples treated with high dosage, viable *E. coli* in ST samples treated with high dosage, and the viable and total *E. coli* in ST samples without chlorination. No significant differences were found in the decay rates between effluent types, bacterial physiological states or chlorine dosages (*p* > 0.05, paired *t*‐test). Nonetheless, the decay rates between different types of FIB were significantly different (*p* < 0.01, paired *t*‐test). The average decay rate of *Enterococcus* was over three times higher than that of *E. coli*.

**TABLE 1:** Decay rate of bacterial populations throughout seawater incubation. First order decay model was applied to calculate the linear regression of concentrations of indicator bacteria (ln cell equivalent or CFU 100 mL^−1^) against time (day), where *k* is the slope (decay rate) and *R* ^2^ is the regression coefficient. Positive value of *k* means decreasing concentrations and negative value means increasing concentrations.

Treatment facility	Indicator bacteria	Population	*k* (N)	*R* ^2^ (N)	*k* (LD)	*R* ^2^ (LD)	*k* (HD)	*R* ^2^ (HD)
SC	*Escherichia coli*	Total	0.50	0.69	0.97	0.84	0.63	0.94
		Viable	0.63	0.85	0.49	0.93	0.54	0.92
		Culturable	0.71	0.85	0.35	0.66	−0.48	0.69
	*Enterococcus*	Total	0.77	0.87	0.73	0.97	0.97	0.93
		Viable	1.19	0.99	1.06	0.96	1.58	0.94
		Culturable	1.04	0.82	0.75	0.59	0.04	0.22
ST	*Escherichia coli*	Total	−0.19	0.51	0.15	0.58	0.53	0.78
		Viable	−0.21	0.58	0.26	0.76	−0.04	0.11
		Culturable	0.39	0.97	ND	ND	ND	ND
	*Enterococcus*	Total	0.54	0.98	0.54	0.82	0.50	0.76
		Viable	2.95	0.88	1.90	0.60	1.59	0.60
		Culturable	1.27	0.79	ND	ND	ND	ND

Abbreviations: CFU, colony forming unit; HD, high chlorine dosage; LD, low chlorine dosage; N, no chlorine; ND, not determined.

In addition, the total bacteria concentrations were around 8–10 log_10_ gene copies mL^−1^ before chlorination in the effluents of SC and ST (Figure S1). Their concentrations reduced by 1–2 log_10_ after chlorination. Higher chlorine dosages resulted in a greater reduction in both total and viable populations. Except for the total populations in SC samples without chlorination, the concentrations of the bacterial load during incubation were similar to those in the seawater control, around 7 and 6 log_10_ gene copies mL^−1^ for the total and viable populations, respectively.

### Alterations in bacterial community composition and diversity

The amplicon sequencing of 240 samples, including PMA‐ and non‐PMA‐treated samples, resulted in an average of 75515.70 ± 14710.36 reads per sample after quality filtering. All samples had >90% of bases with quality score over 30 (Q30). A total of 45,991 sequence variants and 5362 taxa were observed. The 15 most abundant genera in each sewage treatment work were shown in Figure S2. In the SC effluents, *Arcobacter* was the most abundant genus before chlorination, occupying an average of 63.53% of the total annotated genera. Its relative abundances reduced to 49.30% after low dosage and 14.49% after high dosage of chlorine. After 72 h of incubation in seawater, the relative abundances of *Arcobacter* reduced to 10.66 and 1.84% for low and high dosages, respectively. For ST effluents, the most abundant genus before chlorination was *Mycobacterium*, accounting for an average of 13.43% of the total genera. *Mycobacterium* is a type of CRB. Its relative abundance increased to 18.29% after low dosage of chlorine and 31.56% after high dosage of chlorine. However, the relative abundances decreased rapidly to below 0.1% after 48 h in the seawater microcosm regardless of the dosages.

**FIGURE 2:**
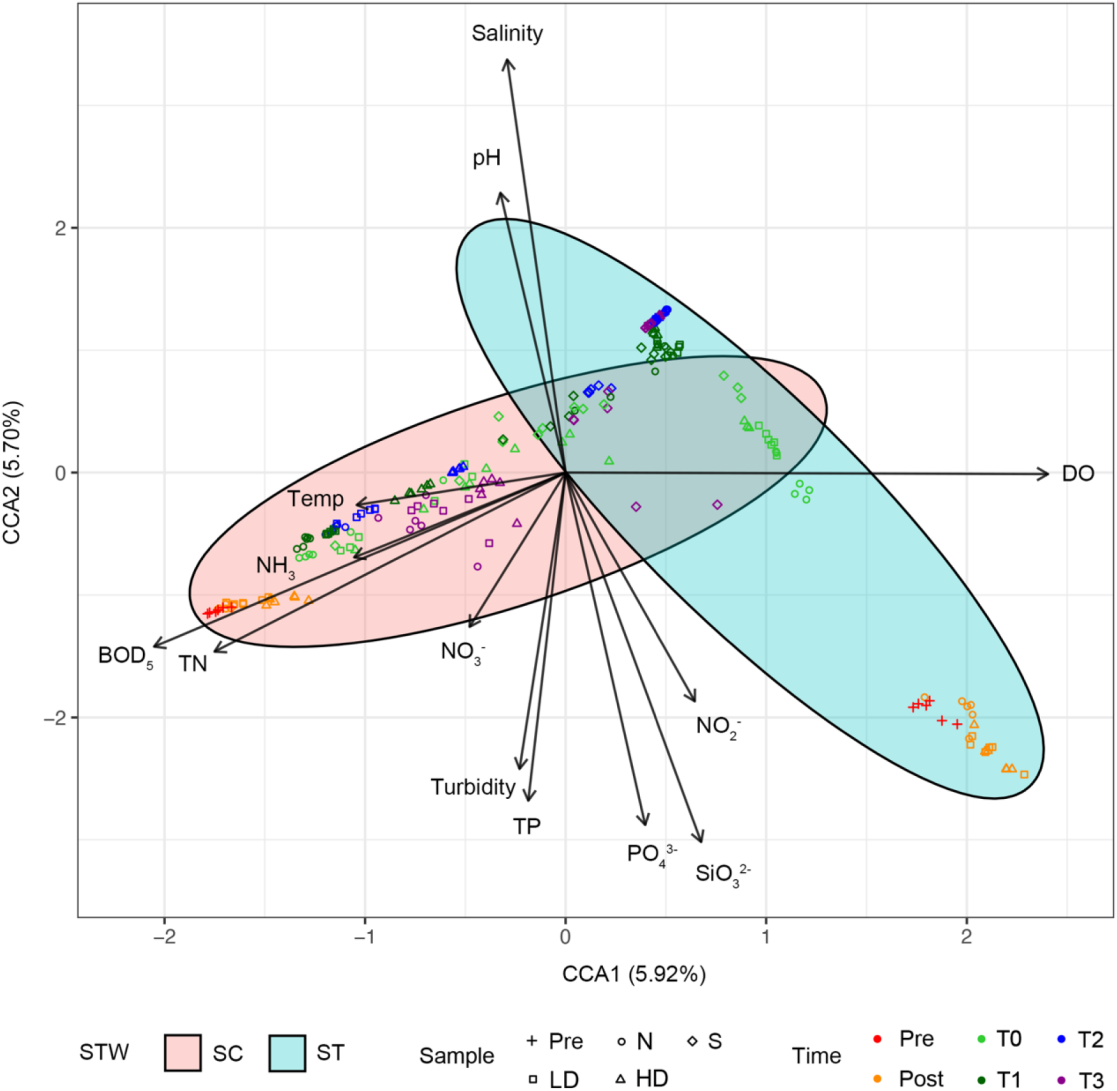
Constrained correspondence analysis (CCA) plot showing the correlations between bacterial community structures and physicochemical parameters. The Chi‐squared distance matrices of sequence variant compositions are explained by the physicochemical parameters. All measured physicochemical parameters are significant constraining variables (*p* < 0.05, PERMANOVA). The direction of arrow indicates the increase in amount of the variable, and the length of arrow is relative to the strength of correlation with the variable. The percentage of variation explained by each constrained axis is indicated. Pre and post indicate samples before and after chlorination, respectively. T0 indicates samples upon dilution with seawater, T1–T3 indicate samples taken from the microcosms at 24, 48 and 72 h. HD, high chlorine dosage; LD, low chlorine dosage; N, no chlorine; S, seawater control.

The bacterial communities before dilution with seawater in SC and ST effluents segregated into two distinct clusters in the CCA plot, in which the SC effluents before dilution were strongly correlated to BOD_5_ and TN (Figure 2). ST samples after seawater dilution clustered much further away from those before dilution but closer to the SC samples. There was no distinct separation between samples of different chlorine dosages at the same time point.

The alpha‐diversity indices (number of sequence variants, Shannon index and Pielou's Evenness index) representing the richness, diversity and evenness of the bacterial communities exhibited a similar pattern over time under different dosage conditions (Figure S3). The three indices of SC samples tended to decrease after 24 h of incubation then increased, while those of ST samples decreased rapidly in 48 h and remained relatively stable afterwards. All the indices of ST samples were significantly lower after 72 h of incubation (Benjamini‐Hochberg adjusted *p* value <0.05, Kruskal–Wallis test).

### SourceTracker analysis of the decay of sewage‐associated genera in seawater

**FIGURE 3:**
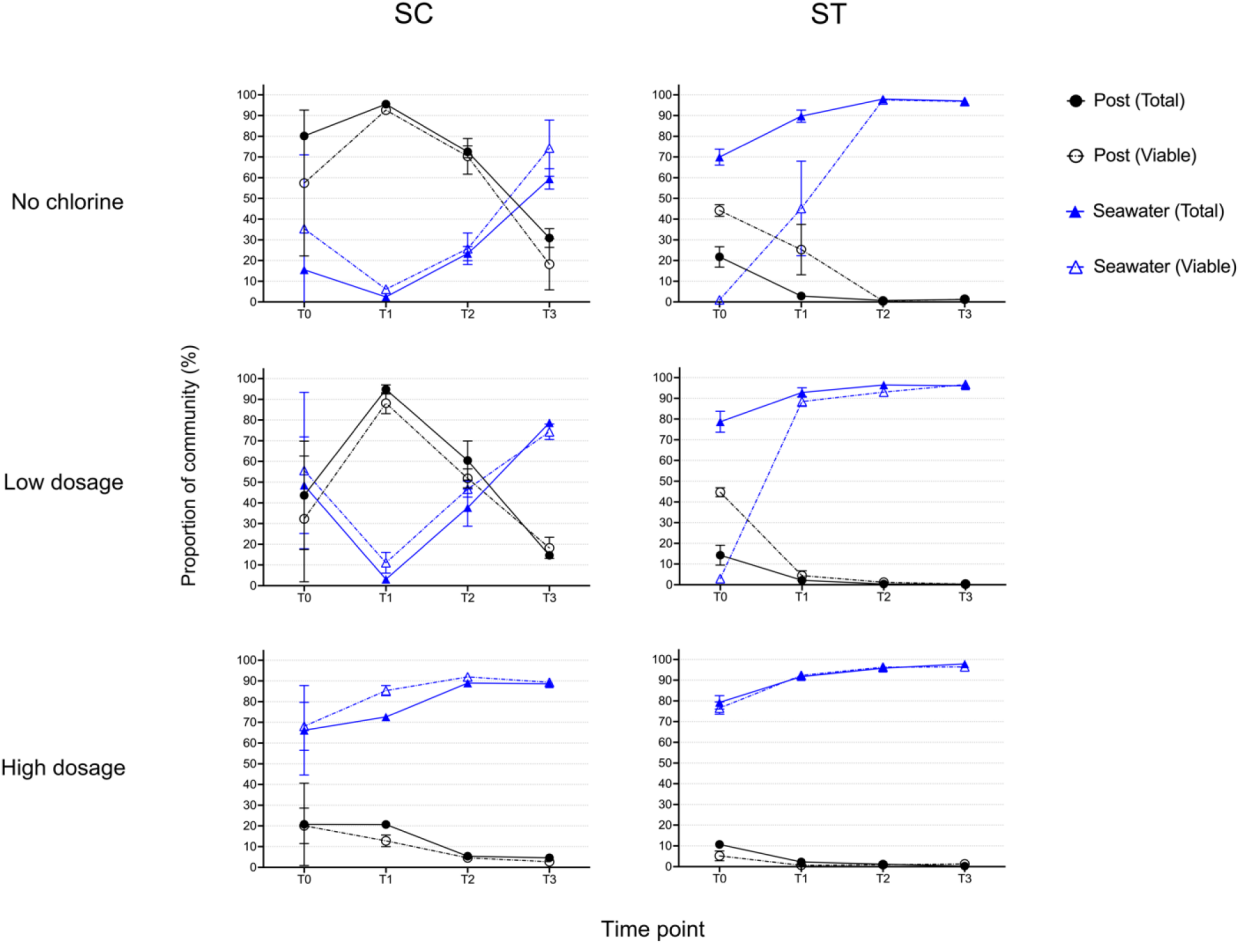
Proportions of effluent and seawater communities in the effluent‐seawater mixtures during incubation. SourceTracker analysis was utilised to evaluate the total and viable proportions of effluents communities after disinfection treatments (post) and seawater controls communities (Seawater) in the effluent‐seawater mixtures of SC and ST for each dosage condition. The genus counts in each samples were used for the analysis. Solid lines and symbols indicate total populations, while dotted lines and hollow symbols indicate viable populations. T0 indicates samples upon dilution with seawater, T1–T3 indicate samples taken from the microcosms at 24, 48 and 72 h. Data shown are mean ± 1 S.D. percentages.

The proportions of effluent communities and seawater communities in the effluent‐seawater mixtures over the period of incubation were determined using SourceTracker (Figure 3). Without chlorination, the total and viable bacterial communities of the SC effluents accounted for 80.15 ± 1.99 and 57.43 ± 35.22% of the whole bacterial communities, respectively at T0, while the total and viable seawater communities accounted for 15.55 ± 0.77 and 35.42 ± 35.62%, respectively. The average percentages of the total and viable effluent communities increased to over 90% after 24 h of incubation but started to decrease afterwards. The total and viable percentages of effluent communities reduced to 30.89 ± 4.54 and 18.24 ± 12.37%, respectively at 72 h of incubation. In contrast, the proportions of total and viable seawater communities increased to 59.45 ± 4.90 and 74.25 ± 13.59%, respectively after 72 h.

For low dosage, the percentages of SC effluent communities were lower than that of the seawater communities at T0. The average percentages of total and viable effluent communities were 43.65 ± 26.18 and 32.25 ± 30.38% at T0, respectively. However, they increased to 94.80 ± 2.19 and 88.12 ± 5.10% after 24 h. Similar to the trend in the samples without chlorination, the percentages of effluent communities reduced afterwards. The total and viable percentages of effluent communities decreased to 14.70 ± 1.62 and 18.29 ± 5.13%, respectively at 72 h, while that of seawater communities increased to 78.68 ± 2.03 and 74.31 ± 3.74%, respectively.

However, the trends of variations in the high dosage of SC effluents were different from the other two conditions. The percentages of seawater communities were over 60% at T0, while that of the effluent communities were only around 20%. The percentages of seawater communities increased throughout the incubation, reaching over 88% after 72 h. Meanwhile, the percentages of effluent communities decreased over time to 4.62 ± 0.37% of the total communities and 2.71 ± 0.44% of the viable communities after 72 h. The viable effluent communities decreased more rapidly in the first 24 h of incubation compared to the total populations.

For ST, the proportions of the effluent communities decreased over time for all three conditions. The percentages of viable effluent communities at T0 were around 44% for no chlorine and low dosage; for high dosage, the percentages of viable effluent communities were only 5.19 ± 2.37% at T0. The percentages of both total and viable effluent communities of the three conditions reduced to around 1% after 48 h of incubation. It is noted that the viable seawater communities in samples were only 1%–2% at T0 for no chlorine and low dosage, and over half of the comprising taxa in the samples were not determined as source of the effluent or seawater communities. Nonetheless, the seawater communities of the three conditions increased in percentages over time, reaching over 95% after 72 h.

**FIGURE 4:**
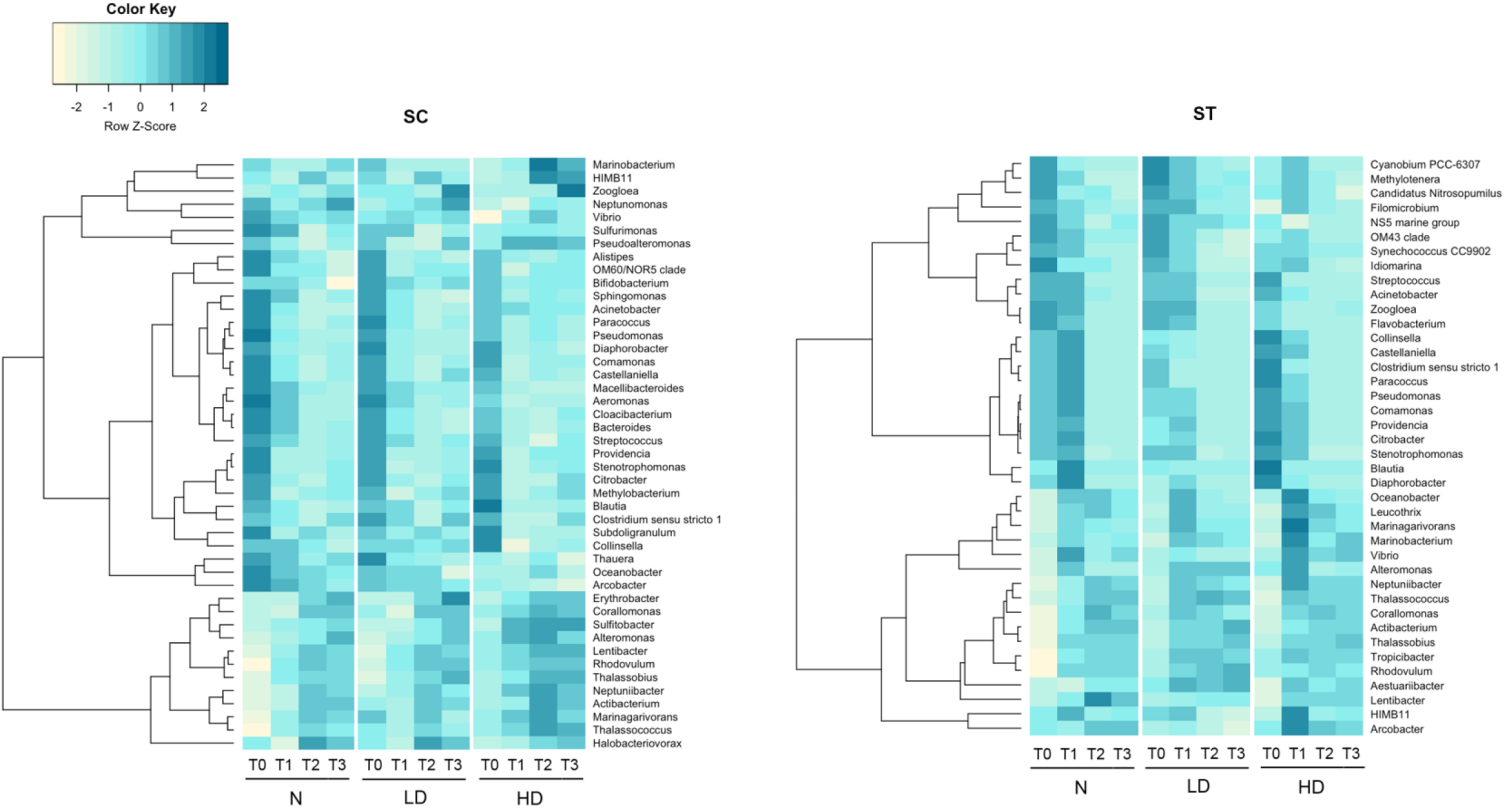
Viable concentrations of the genera from effluent communities during incubation in the microcosms. Genera originated from the source of disinfected effluent communities were classified by SourceTracker. Those with relative abundance over 0.01% and common in both SC and ST samples were shown in the heatmaps. The concentrations were averaged over viable triplicates. Colour scale represented the *Z*‐scores of the concentrations in rows. T0 indicates samples upon dilution with seawater, T1–T3 indicate samples taken from the microcosms at 24, 48 and 72 h. HD: high chlorine dosage; LD: low chlorine dosage; N: no chlorine.

Numerous genera in the effluent‐seawater mixtures were classified as originating from the source of effluent communities by SourceTracker (Figure 4). Their relative abundances had been normalised by the 16S rRNA copies of the total bacterial load for estimating the concentrations. Considering the viable genera with relative abundances above 0.01% and those that were commonly found in both SC and ST samples, 45 genera from SC effluents and 40 genera from ST effluents were identified (Figure 4). The average concentrations of 26 genera in SC samples and 22 genera in ST samples decreased after 72 h of incubation for all three conditions. The average reduction of these genera was 2.19 ± 1.20 and 3.05 ± 1.42 log_10_ mL^−1^ in SC and ST samples, respectively. Genera including *Castellaniella*, *Pseudomonas*, *Comamonas* and *Diaphorobacter* reduced in both SC and ST samples. Yet, some genera had opposite trends of variation in SC and ST samples. For example, *Arcobacter* were enriched after incubation in ST samples, but reduced in SC samples.

Regarding the rapid increase in the percentages of SC effluent communities at T1 for no chlorine and low dosage (Figure 3), it was observed that *Lentibacter* and *Rhodovulum* had the largest increase in concentrations for both conditions (*p* < 0.05, *t*‐test). Their concentrations increased over 2 log_10_ mL^−1^ after 24 h of incubation. Including *Lentibacter* and *Rhodovulum*, several genera such as *Sulfitobacter*, *Zoogloea* and *Erythrobacter* increased over 3 log_10_ mL^−1^ after 72 h of incubation.

Many genera in ST samples decayed faster during the incubation than those in SC. The concentrations of the genera in ST and SC samples at T0 were similar, around 3–4 log_10_ mL^−1^ on average. In SC samples, all the genera were still present after 72 h of incubation. However, genera in ST samples including *Flavobacterium*, *Comamonas*, *Citrobacter*, *Castellaniella*, *Streptococcus*, *Paracoccus*, *Providencia*, *Clostridium* *sensu stricto* *1* and *Collinsella* became undetectable after 48 h for all the dosage conditions. The average reduction of their concentrations was over 3 log_10_ mL^−1^.

### Survival of CRB during chlorination and seawater incubation

**FIGURE 5:**
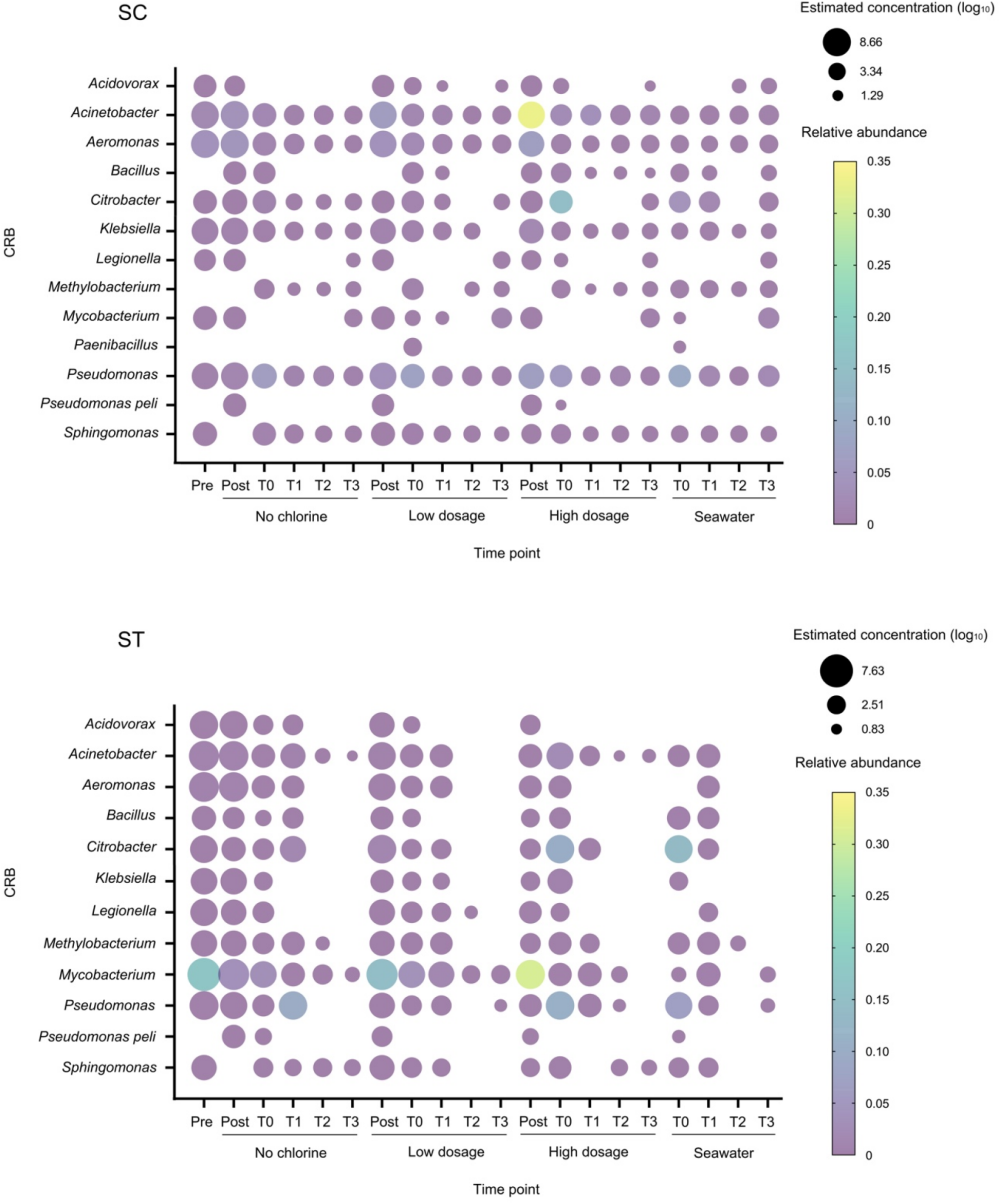
Alterations of the relative abundances and concentrations of viable chlorine‐resistant bacteria (CRB) under different chlorine dosages and during seawater incubation. Viable chlorine‐resistant genera and species identified in the amplicon sequences of SC and ST samples are shown. Estimated concentrations of the CRB are represented by the size of bubbles. The bubble sizes of the largest value, geometric mean of the smallest and largest value, and the smallest value in the data set are shown in the legend. Relative abundances of the CRB are represented by the colour scale. Pre and post indicate samples before and after chlorination, respectively. T0 indicates samples upon dilution with seawater, T1–T3 indicate samples taken from the microcosms at 24, 48 and 72 h.

Typical CRB were identified in the amplicon sequences of the PMA‐treated samples (Figure 5). In total, 13 types of viable CRB were found. Many of them were significantly enriched after chlorination, especially under high dosage (*p* < 0.05, *t*‐test). The relative abundances of many CRB increased with the dosage of chlorine. For example, the average relative abundance of *Acinetobacter* in SC samples after low dosage was 6.09 ± 3.26%, which was 3.19 fold of its relative abundance before chlorination. Under high dosage, the abundance reached 33.14 ± 2.33%, which was 17.35 fold of the abundance before chlorination. The relative abundance and fold change of *Acinetobacter* after high dosage in SC samples were the highest among all samples. For ST samples, *Mycobacterium* after high dosage of chlorine had the highest relative abundance among all the CRB, reaching over 30% on average.

The average concentrations of CRB before chlorination in SC samples were 6.83 ± 1.09 log_10_ 16S rRNA copies mL^−1^, while that in ST samples were 5.42 ± 0.93 log_10_ copies mL^−1^ (neglecting those that were absent before chlorination). The concentrations of *Aeromonas* and *Acinetobacter* were the highest in SC samples before chlorination, both over 8 log_10_ copies mL^−1^. While in ST samples, the concentration of *Mycobacterium* was the highest (over 7 log_10_ copies mL^−1^). Except for *Pseudomonas* and *Pseudomonas peli*, all the CRB in SC samples reduced in concentration after low and high dosages of chlorine. The average reduction of CRB in SC samples under low dosage was 0.31 ± 0.23 log_10_ copies mL^−1^, while that under high dosage was 0.90 ± 0.46 log_10_ copies mL^−1^. For ST samples, all the CRB reduced in concentration after low and high dosages except *P. peli* and *Citrobacter*. The average log_10_ reductions of CRB in ST samples were 0.79 ± 0.36 and 2.70 ± 0.33 under low and high dosages, respectively.

Upon incubation in the seawater microcosm, most of the CRB exhibited a reduction in their relative abundances in the first 24 h. In SC samples, the relative abundances of *Aeromonas*, *Bacillus*, *Citrobacter*, *Klebsiella*, *Methylobacterium*, *Pseudomonas* and *Sphingomonas* reduced after 24 h for all three dosage conditions. In addition to the CRB mentioned above, *Acinetobacter* and *Legionella* also reduced for low and high dosages in ST samples after 24 h of incubation. Although some of the relative abundances increased during the incubation period, the average relative abundances of all these CRB reduced for all three dosage conditions after 72 h in both SC and ST samples. The reduction of relative abundances over the incubation period did not have significant difference between dosage conditions (*p* > 0.05, paired *t*‐test).

The concentrations of CRB in the seawater microcosm diminished more rapidly in ST than SC samples, most of the CRB in ST samples were barely detected after 48 h. For those CRB persisting after 72 h of incubation, their average concentrations ranged from 1.30 to 4.63 log_10_ copies mL^−1^ in SC samples and 0.83 to 2.53 log_10_ copies mL^−1^ in ST samples. No significant difference in the concentrations of CRB was found between different dosage conditions or sewage treatment works after 72 h (*p* > 0.05, paired *t*‐test). It is noted that the average concentrations of these persisting CRB for the three dosage conditions did not have significant difference with the seawater control. In fact, considerable number of CRB were present in the seawater control during the whole incubation period. The average concentration of CRB existing in the seawater control during the incubation period was around 3 log_10_ copies mL^−1^.

**FIGURE 6:**
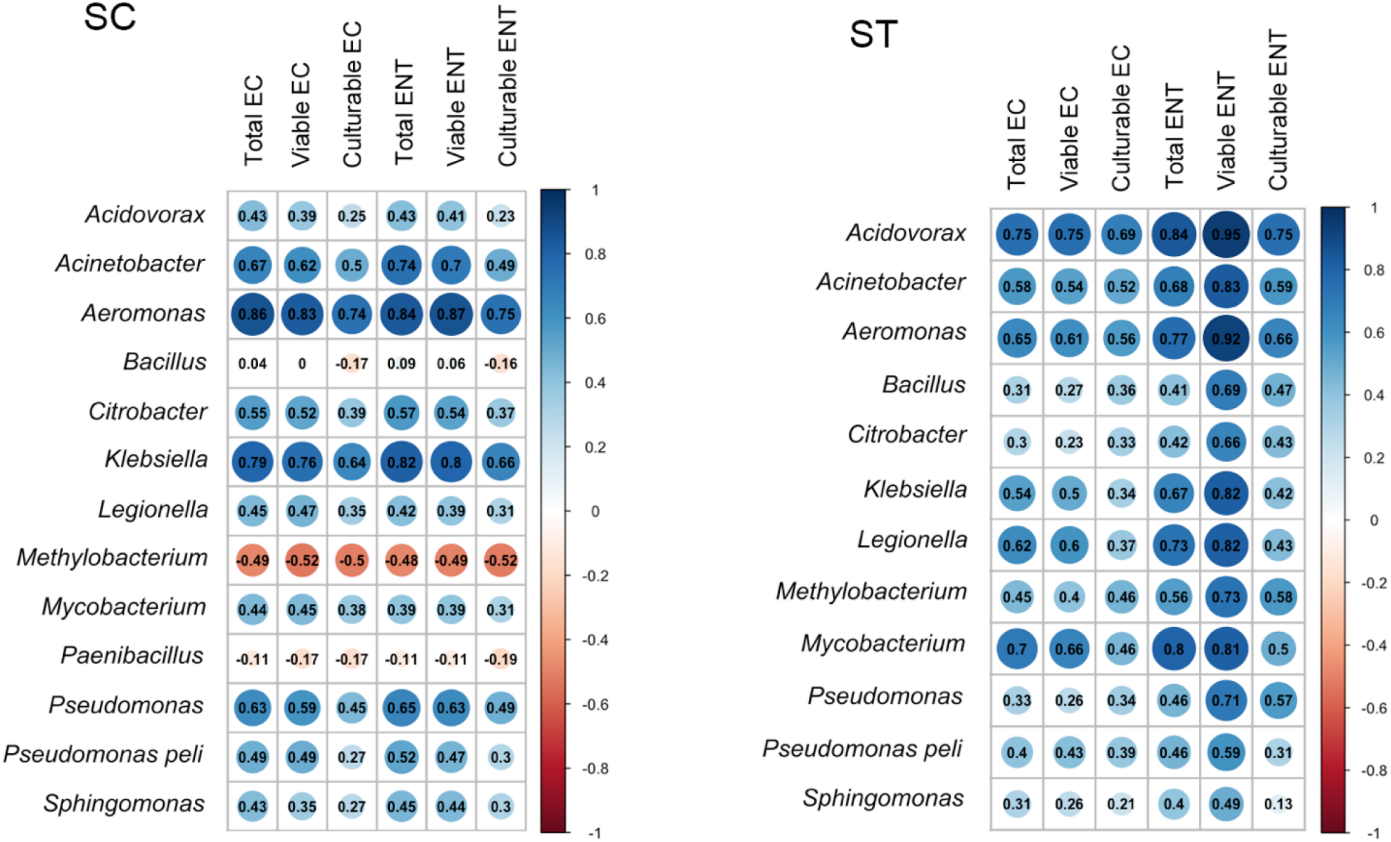
Spearman's rank correlations between the concentrations of CRB and indicator bacteria. The correlations between the viable concentrations of CRB and the total, viable and culturable concentrations of *Escherichia coli* (EC) and *Enterococcus* (ENT) in SC and ST samples are shown. The range of correlation is depicted by the size and colour of bubbles, while the numbers indicate the values of correlation coefficient.

Specific CRB exhibited strong correlations with the total, viable and culturable concentrations of *E. coli* and *Enterococcus*, such as *Aeromonas* in SC and *Acidovorax* in ST (*r*_s_ ≥0.7, Spearman's rank correlation) (Figure 6). However, strong correlation in one type of effluent did not necessarily occur in another. For example, *Acidovorax* exhibited strong correlations with the FIB in ST samples but weak correlations in SC samples. Genera such as *Bacillus* and *Paenibacillus* had very weak or even no correlation with different populations of FIB (| *r*_s_ | < 0.2). Although the strength of correlations varied with different CRB and effluent types, it is observed that most of the culturable concentrations of FIB had weaker correlations than the total and viable concentrations. Comparing the correlation strengths of the two FIB, the viable concentrations of *Enterococcus* had relatively strong correlations with most of the CRB, especially in ST samples (*r*_s_ >0.7).

## DISCUSSION

### Survival of FIB in aquatic environment

FIB are used as proxies to evaluate disinfection outcomes in sewage treatment works. Therefore, their physiological states, disinfectant resistance and environmental persistence are major concerns in effluent monitoring. In this study, the physiological states of two conventional FIB, *E. coli* and *Enterococcus*, were traced throughout the disinfection process and simulated discharge into the receiving environment. There was a large discrepancy between the viable and culturable populations of FIB, which widened with the increase of chlorine dosages (Figure 1). The discrepancy can be attributed to the ability of bacteria to enter VBNC states under sublethal stress, which in this case was the oxidative stress of chlorine. This raises the question of whether surviving viable FIB, which cannot be detected in conventional culture‐based enumeration methods, thrive and resuscitate into reproductive culturable forms after alleviation of the chlorine stress.

In our results, the total, viable and culturable populations of FIB tended to decrease after dilution and incubation in seawater (Figure 1 & Table 1). Such decline of FIB in seawater was also observed in previous studies (Noble et al., 2004; Solecki et al., 2011). Interestingly, the decrease of populations in seawater incubation did not have significant difference between samples with or without chlorination (*p* > 0.05, paired *t*‐test). This suggests that the ability of FIB to survive in the natural environment did not weaken due to the sublethal dosage of chlorine. Particularly, it is observed that the culturable populations of *E. coli* under high dosage in SC samples slightly rose after 72 h of incubation, although the rise could not be concluded as resuscitation of VBNC cells or growth of the persistent culturable cells. On the other hand, no sight of resuscitation or regrowth of culturable cells was observed in other sets of experiment. In fact, resuscitation of VBNC cells may not easily occur in seawater. Even in culture medium, attempts to achieve resuscitation of VBNC bacteria were not successful in some studies (Chen et al., 2018; Oliver et al., 2005; Özkanca et al., 2009). These studies pointed out several criteria for resuscitation, such as nutrient‐rich conditions, uplifting of temperature and additions of beneficial chemicals. Furthermore, since *E. coli* and *Enterococcus* are not indigenous to seawater, conditions such as salinity, pH and microbiota exert pressure on the FIB (Wanjugi & Harwood, 2013). Therefore, these FIB died off or persisted in the VBNC state during seawater incubation. However, it should be noted that the persistence of VBNC bacteria in seawater suggests a potential threat to public health. Pathogens in the faecal sources might also enter VBNC states under chlorine stress. When these VBNC pathogens enter host animals, they can resuscitate and become infectious (Fakruddin et al., 2013).

In this study, *Enterococcus* had significantly higher decay rate in seawater than *E. coli* (Table 1). This finding contradicts most others, in which *Enterococcus* was more resistant than *E. coli* in seawater (Byappanahalli et al., 2012; WHO, 1999; Wong et al., 2022). Furthermore, *Enterococcus* has been recommended for use as an indicator of seawater for years (USEPA, 2003; WHO, 1999). However, unlike many regulatory agencies around the world, the Hong Kong Government does not apply *Enterococcus* as a water quality indicator in current monitoring regime. The government has organised various scientific studies on the potential of *Enterococcus* being a suitable indicator in the local context (Thoe et al., 2018). The reports indicated that *Enterococcus* in local beach water had very low abundance (consistently at 2–9 CFU 100 mL^−1^) and weaker correlations with disease rates than *E. coli*. The exact reason is yet to be concluded but it was found that the populations of *E. faecalis* and *E. faecium*, two major *Enterococcus* species in local faecal sources, were very genetically diverse. The great variance in genetic makeup may cause heterogeneous survival rates of *Enterococcus* in seawater, as some *Enterococcus* strains appeared to have higher decay rates than *E. coli* (Anderson et al., 2005; Lleò et al., 2005). This indicates the importance of conducting local studies in the selection of FIB for water quality monitoring.

### Dynamics of the sewage‐associated bacterial communities and their implications to the environment

At the community level, the bacteria in the treated effluents of two sewage treatment works before chlorination differed substantially in community structures and compositions (Figure 2 & Figure S2). Comparing to SC, ST effluents had much lower BOD_5_ and TN concentrations due to the consumption of organic matter and nutrients in the biomass during secondary treatment. As a result, SC samples tended to cluster at high BOD_5_ and TN. Among the top 15 abundant bacteria in each type of effluent, it was observed that chlorination enriched specific types of bacteria in the communities, especially for those with high chlorine resistance such as *Acinetobacter* and *Mycobacterium* (Figure S2). Their relative abundances increased with higher dosages.

During the seawater incubation, we found that the proportions of effluent bacterial communities exhibited distinct patterns among different chlorine dosage conditions and effluent types (Figure 3). First, higher chlorine dosage reduced the percentages of total and viable effluent communities at T0 and their survival in the seawater afterwards. After treating with high dosage of chlorine, the effluent communities had much lower proportions in the community than the seawater communities throughout the incubation period. The percentages of effluent communities diminished to nearly 0% after 72 h of incubation in both treatment works. High dosage of chlorine was highly effective in reducing the disruption of effluent bacterial communities to the indigenous seawater communities.

Second, the trends of the proportions of effluent communities during incubation were different between SC and ST samples. Instead of reducing gradually in ST samples, the percentages of effluent communities without chlorination and under low dosage in SC samples showed a rapid increase at 24 h (Figure 3). They reached around 90% for both total and viable communities. Moreover, many sewage‐associated genera diminished rapidly during the incubation in ST samples but not in SC samples (Figure 4). The reason for the difference in the survival trends may be attributed to the influent type. Since the influent in SC was seawater‐based, the bacterial communities established in SC effluents should be more adapted to the saline environment. Many bacteria from SC effluents were commonly found in seawater, such as *Rhodovulum*, *Sulfitobacter* and *Erythrobacter* (Kikuchi & Umekage, 2018; Kim et al., 2022; Yoon et al., 2004). These bacteria showed rapid growth during incubation in seawater (Figure 4). The quenching of chlorination and dilution with seawater might be favourable for their growth as they were released from the oxidative stress of chlorine and returned to a saline environment. In contrast, the influent in ST was freshwater‐based, the bacterial communities encountered stress from the high salinity of seawater, thus their regrowth was suppressed.

These observations imply that factors affecting the survival of effluent communities in the receiving environment depend not only on the environmental conditions but also the upstream treatment processes prior to discharge, such as the dosage of chlorine. High dosage can reduce the percentages of effluent communities and the chance of bacterial regrowth in seawater. The rebound of effluent communities in SC was not observed for high dosage, it could be due to the low amount of effluent bacteria at the beginning of incubation and the slow recovery from the strong oxidative stress. Furthermore, the survival trends of effluent communities can differ between sewage treatment works. It may be due to the distinctive bacterial community compositions established in different types of effluent. Bacterial communities from saline‐based effluents may have better survival in saline environment.

Nonetheless, our study showed that the indigenous seawater bacterial communities had strong resilience after exposing to effluents. The sewage‐associated genera did not persist in the microcosms at the end of incubation (Figure 3). The seawater communities recovered over time and dominated the bacterial microbiome after 72 h under all experimental conditions. They could regrow rapidly within a few days even though their proportions had been reduced to a low level (<10%) after the perturbation of effluents. Further study on the required time for recovering to the pre‐disturbance level under different scenarios and the stability of the recovery of microbiome will be valuable for environmental risk management.

It is noted that the proportions of viable seawater communities were typically low in the ST samples at T0 for the treatments with no chlorine and low dosage. Around half of the population could not be assigned to either effluent or seawater source (Figure 3). With reference to the genera of unknown source in those samples, many of them were common among the samples. Some of them were ubiquitous prokaryotes living in the marine environment, such as SAR11 clades (*Clade Ia, Ib and II*), *Nitrosopumilus*, *NS5 marine group*, *Actinomarina*, and so forth. SAR11 clade (*Clade Ia*) and *Candidatus Nitrosopumilus* were the two most abundant genera in these samples, accounting for around 20%–30% of the genera of unknown source. These genera might originate from the seawater used for dilution. However, these genera were not found in the water control at T0. One possibility is the microbial communities in the continuous flow of seawater supply from the coastal pier changed during the processing of samples of no chlorine or low dosage at T0, therefore a considerable proportion of viable seawater communities in the samples could not be assigned to the seawater source.

### Fate of CRB after chlorine disinfection and effluent discharge

The chlorine‐resistant genera investigated in this study were widely observed in effluent and water distribution systems. Many of them were opportunistic pathogenic bacteria (Luo et al., 2021) (Figure 5). They were enriched after chlorination. Higher dosages of chlorine appeared to strengthen the selective force for CRB in the community. The relative abundances of *Acinetobacter* and *Mycobacterium* reached over 30% in the bacterial communities after being treated with high dosage of chlorine. Despite of the increase in relative abundances, the estimated concentrations of these CRB reduced after chlorination. Most of them were further reduced during incubation in seawater. The dosage conditions did not have significant effect on the survival of CRB in seawater.

The correlations between the concentrations of CRB and FIB showed the limitations of conventional FIB in representing the quantities of these genera (Figure 6). One of the issues is the large variation in the correlation strength with different CRB. The concentrations of CRB throughout chlorine disinfection and seawater incubation varied greatly between different genera (Figure 5). Such variations led to different strengths of correlation between CRB and FIB. Certain CRB had particularly weak correlations with the concentrations of *E. coli* and *Enterococcus*, regardless of the physiological states. Even though some CRB might show strong correlations with specific physiological states of FIB, the strength changed with the types of effluents. Although the viable concentrations of *Enterococcus* had strong correlations with most of the CRB in ST effluents, this condition cannot be generalised to all types of effluents.

Most importantly, culturable plate counts of *E. coli* and *Enterococcus*, which are commonly used standards for determining the effluent disinfection outcomes, were weak in tracing the CRB. It is because large proportions of viable FIB were unculturable, especially under high dosage of chlorine (Figure 1). Using culturable counts of FIB will underestimate the survival of FIB in the effluents and thus weaken their correlations with the viable concentrations of CRB. Comparatively, the total and viable gene copies of FIB had stronger correlations with most of the CRB in effluents. It showed that the qPCR quantification of gene copies is more reliable than bacterial cultivation in assessing the presence of CRB through FIB.

## CONCLUSION

Increasing the dosage of chlorine disinfection could effectively reduce the disruption of effluent bacterial communities to the indigenous seawater communities after discharge. Bacterial communities from saline‐based effluents exhibited stronger survival in seawater, thus higher chlorine dosage was required to suppress their regrowth. However, it is important to note that higher dosages induced more FIB into the VBNC state and enriched the pathogenic CRB in the communities. Conventional culture‐based enumeration methods of FIB were not adequate to trace the CRB. The qPCR quantification of gene markers of FIB showed stronger correlations with the presence of CRB, although the strength varied between different types of effluents. Therefore, it is crucial to consider the intrinsic properties of effluents together with the properties of the receiving environment in the design of disinfection regimes and monitoring strategies.

## AUTHOR CONTRIBUTIONS

**Mandy Lok Yi Tang:** Conceptualization (equal); data curation (equal); formal analysis (equal); investigation (equal); methodology (equal); software (equal); validation (equal); visualization (equal); writing – original draft (equal). **Stanley Chun Kwan Lau:** Conceptualization (equal); data curation (equal); funding acquisition (equal); methodology (equal); project administration (equal); resources (equal); supervision (equal); validation (equal); writing – review and editing (equal).

## CONFLICT OF INTEREST STATEMENT

The authors declare no conflict of interest.

## Supporting information


**DATA S1:** Supporting Information.

## Data Availability

The 16S rRNA amplicon sequencing data of 240 samples were deposited at NCBI Sequence Read Archive (SRA) with accession no. of PRJNA972297.

## References

[emi413216-bib-0001] Acharya, K. , Halla, F.F. , Massawa, S.M. , Mgana, S.M. , Komar, T. , Davenport, R.J. et al. (2020) Chlorination effects on DNA based characterization of water microbiomes and implications for the interpretation of data from disinfected systems. Journal of Environmental Management, 276, 111319. 32889498 10.1016/j.jenvman.2020.111319

[emi413216-bib-0002] Ahmed, W. , Staley, C. , Kaiser, T. , Sadowsky, M.J. , Kozak, S. , Beale, D. et al. (2018) Decay of sewage‐associated bacterial communities in fresh and marine environmental waters and sediment. Applied Microbiology and Biotechnology, 102, 7159–7170. 29869677 10.1007/s00253-018-9112-4

[emi413216-bib-0003] Anderson, K.L. , Whitlock, J.E. & Harwood, V.J. (2005) Persistence and differential survival of fecal indicator bacteria in subtropical waters and sediments. Applied and Environmental Microbiology, 71, 3041–3048. 15933000 10.1128/AEM.71.6.3041-3048.2005 PMC1151827

[emi413216-bib-0004] APHA . (2005) Standard methods for the examination of water and wastewater, 21st edition. Washington: American Public Health Association.

[emi413216-bib-0005] Bolyen, E. , Rideout, J.R. , Dillon, M.R. , Bokulich, N.A. , Abnet, C.C. , Al‐Ghalith, G.A. et al. (2019) Reproducible, interactive, scalable and extensible microbiome data science using QIIME 2. Nature Biotechnology, 37, 852–857. 10.1038/s41587-019-0209-9 PMC7015180 31341288

[emi413216-bib-0006] Byappanahalli, M.N. , Nevers, M.B. , Korajkic, A. , Staley, Z.R. & Harwood, V.J. (2012) Enterococci in the environment. Microbiology and Molecular Biology Reviews, 76, 685–706. 23204362 10.1128/MMBR.00023-12 PMC3510518

[emi413216-bib-0007] Callahan, B.J. , McMurdie, P.J. , Rosen, M.J. , Han, A.W. , Johnson, A.J.A. & Holmes, S.P. (2016) DADA2: high‐resolution sample inference from Illumina amplicon data. Nature Methods, 13, 581–583. 27214047 10.1038/nmeth.3869 PMC4927377

[emi413216-bib-0008] Chandy, J.P. & Angles, M.L. (2001) Determination of nutrients limiting biofilm formation and the subsequent impact on disinfectant decay. 10.1016/s0043-1354(00)00572-8 11456167

[emi413216-bib-0009] Chen, S. , Li, X. , Wang, Y. , Zeng, J. , Ye, C. , Li, X. et al. (2018) Induction of Escherichia coli into a VBNC state through chlorination/chloramination and differences in characteristics of the bacterium between states. Water Research, 142, 279–288. 29890476 10.1016/j.watres.2018.05.055

[emi413216-bib-0010] Choi, K.W. , Lee, J.H.W. , Kwok, K.W.H. & Leung, K.M.Y. (2009) Integrated stochastic environmental risk assessment of the Harbour Area Treatment Scheme (HATS) in Hong Kong. Environmental Science & Technology, 43, 3705–3711. 19544877 10.1021/es803244s

[emi413216-bib-0011] Cloete, T.E. (2003) Resistance mechanisms of bacteria to antimicrobial compounds. In: International biodeterioration and biodegradation, Vol. 51. Elsevier Ltd, pp. 277–282.

[emi413216-bib-0012] Desmarais, T.R. , Solo‐Gabriele, H.M. & Palmer, C.J. (2002) Influence of soil on fecal indicator organisms in a tidally influenced subtropical environment. Applied and Environmental Microbiology, 68, 1165–1172. 11872464 10.1128/AEM.68.3.1165-1172.2002 PMC123749

[emi413216-bib-0013] Fakruddin, M. , Mannan, K.S.B. & Andrews, S. (2013) Viable but nonculturable bacteria: food safety and public health perspective. International Scholarly Research Notices, 2013, 1–6. 10.1155/2013/703813 PMC3804398 24191231

[emi413216-bib-0014] Furuhata, K. , Ishizaki, N. , Umekawa, N. , Nishizima, M. & Fukuyama, M. (2014) Pulsed‐field gel electrophoresis pfge pattern analysis and chlorine‐resistance of *Legionella pneumophila* isolated from hot spring water samples. 10.4265/bio.19.33 24670616

[emi413216-bib-0015] Ho, B.S.W. & Tam, T.‐Y. (1997) Enumeration of *E. coli* in environmental waters and wastewater using a chromogenic medium. Water Science and Technology, 35, 409–413.

[emi413216-bib-0016] Jian, C. , Luukkonen, P. , Yki‐Järvinen, H. , Salonen, A. & Korpela, K. (2020) Quantitative PCR provides a simple and accessible method for quantitative microbiota profiling. PLoS One, 15, e0227285. 31940382 10.1371/journal.pone.0227285 PMC6961887

[emi413216-bib-0017] Kikuchi, Y. & Umekage, S. (2018) Extracellular nucleic acids of the marine bacterium *Rhodovulum sulfidophilum* and recombinant RNA production technology using bacteria. FEMS Microbiology Letters, 365(3). 10.1093/femsle/fnx268 29228187

[emi413216-bib-0018] Kim, M. , Cha, I.T. , Lee, K.E. & Park, S.J. (2022) Sulfitobacter albidus sp. nov., isolated from marine sediment of Jeju Island. Archives of Microbiology, 204(12), 691. 36334148 10.1007/s00203-022-03305-x

[emi413216-bib-0019] Knights, D. , Kuczynski, J. , Charlson, E.S. , Zaneveld, J. , Mozer, M.C. , Collman, R.G. et al. (2011) Bayesian community‐wide culture‐independent microbial source tracking. Nature Methods, 8, 761–765. 21765408 10.1038/nmeth.1650 PMC3791591

[emi413216-bib-0020] Korajkic, A. , McMinn, B.R. , Shanks, O.C. , Sivaganesan, M. , Fout, G.S. & Ashbolt, N.J. (2014) Biotic interactions and sunlight affect persistence of fecal indicator bacteria and microbial source tracking genetic markers in the upper Mississippi river. Applied and Environmental Microbiology, 80, 3952–3961. 24747902 10.1128/AEM.00388-14 PMC4054226

[emi413216-bib-0021] Korajkic, A. , Wanjugi, P. & Harwood, V.J. (2013) Indigenous microbiota and habitat influence escherichia coli survival more than sunlight in simulated aquatic environments. Applied and Environmental Microbiology, 79, 5329–5337. 23811514 10.1128/AEM.01362-13 PMC3753954

[emi413216-bib-0022] Li, L. , Mendis, N. , Trigui, H. , Oliver, J.D. & Faucher, S.P. (2014) The importance of the viable but non‐culturable state in human bacterial pathogens. Frontiers in Microbiology, 5, 258. 24917854 10.3389/fmicb.2014.00258 PMC4040921

[emi413216-bib-0023] Lin, H. , Ye, C. , Chen, S. , Zhang, S. & Yu, X. (2017) Viable but non‐culturable *E. coli* induced by low level chlorination have higher persistence to antibiotics than their culturable counterparts. Environmental Pollution, 230, 242–249. 28662489 10.1016/j.envpol.2017.06.047

[emi413216-bib-0024] Liu, R. , Cheng, K.H.F. , Wong, K. , Cheng, S.C.S. & Lau, S.C.K. (2015) Differential utility of the Bacteroidales DNA and RNA markers in the tiered approach for microbial source tracking in subtropical seawater. Applied Microbiology and Biotechnology, 99, 5669–5681. 25652655 10.1007/s00253-015-6410-y

[emi413216-bib-0025] Lleò, M.D.M. , Bonato, B. , Benedetti, D. & Canepari, P. (2005) Survival of enterococcal species in aquatic environments. FEMS Microbiology Ecology, 54, 189–196. 16332318 10.1016/j.femsec.2005.03.016

[emi413216-bib-0026] Luo, L.W. , Wu, Y.H. , Yu, T. , Wang, Y.H. , Chen, G.Q. , Tong, X. et al. (2021) Evaluating method and potential risks of chlorine‐resistant bacteria (CRB): a review. Water Research, 188, 116474. 33039832 10.1016/j.watres.2020.116474

[emi413216-bib-0027] Naidoo, S. & Olaniran, A.O. (2013) Treated wastewater effluent as a source of microbial pollution of surface water resources. International Journal of Environmental Research and Public Health, 11, 249–270. 24366046 10.3390/ijerph110100249 PMC3924443

[emi413216-bib-0028] Noble, R.T. , Lee, I.M. & Schiff, K.C. (2004) Inactivation of indicator micro‐organisms from various sources of faecal contamination in seawater and freshwater. Journal of Applied Microbiology, 96, 464–472. 14962126 10.1111/j.1365-2672.2004.02155.x

[emi413216-bib-0029] Nontaleerak, B. , Duang‐nkern, J. , Wongsaroj, L. , Romsang, A. , Trinachartvanit, W. & Mongkolsuk, S. (2020) Roles of RcsA, an AhpD family protein, in reactive chlorine stress resistance and virulence in *Pseudomonas aeruginosa* . Applied and Environmental Microbiology, 86, 1–22. 10.1128/AEM.01480-20 PMC7531977 32801171

[emi413216-bib-0030] Oliver, J.D. , Dagher, M. & Linden, K. (2005) Induction of *Escherichia coli* and *Salmonella typhimurium* into the viable but nonculturable state following chlorination of wastewater. Journal of Water and Health, 3, 249–257. 16209029 10.2166/wh.2005.040

[emi413216-bib-0031] Özkanca, R. , Saribiyik, F. , Isik, K. , Sahin, N. , Kariptas, E. & Flint, K.P. (2009) Resuscitation and quantification of stressed *Escherichia coli* K12 NCTC8797 in water samples. Microbiological Research, 164, 212–220. 17418553 10.1016/j.micres.2006.11.014

[emi413216-bib-0032] Rochelle‐Newall, E. , Nguyen, T.M.H. , Le, T.P.Q. , Sengtaheuanghoung, O. & Ribolzi, O. (2015) A short review of fecal indicator bacteria in tropical aquatic ecosystems: knowledge gaps and future directions. Frontiers in Microbiology, 6, 308. 25941519 10.3389/fmicb.2015.00308 PMC4400915

[emi413216-bib-0033] Schottroff, F. , Fröhling, A. , Zunabovic‐Pichler, M. , Krottenthaler, A. , Schlüter, O. & Jäger, H. (2018) Sublethal injury and viable but non‐culturable (VBNC) state in microorganisms during preservation of food and biological materials by non‐thermal processes. Frontiers in Microbiology, 9, 2773. 30515140 10.3389/fmicb.2018.02773 PMC6255932

[emi413216-bib-0034] Solecki, O. , Jeanneau, L. , Jardé, E. , Gourmelon, M. , Marin, C. & Pourcher, A.M. (2011) Persistence of microbial and chemical pig manure markers as compared to faecal indicator bacteria survival in freshwater and seawater microcosms. Water Research, 45, 4623–4633. 21745675 10.1016/j.watres.2011.06.012

[emi413216-bib-0035] Su, J. , Fan, J. , Ming, H. , Guo, G. , Fu, Y. , Zhao, X. et al. (2022) The municipal sewage discharge may impact the dissemination of antibiotic‐resistant *Escherichia coli* in an Urban Coastal Beach. Water (Switzerland), 14, 1639.

[emi413216-bib-0036] Tang, M.L.Y. & Lau, S.C.K. (2022) Strategy to evaluate changes in bacterial community profiles and bacterial pathogen load reduction after sewage disinfection. Frontiers in Microbiology, 13, 919207. 35898906 10.3389/fmicb.2022.919207 PMC9309643

[emi413216-bib-0037] Tettamanti Boshier, F.A. , Srinivasan, S. , Lopez, A. , Hoffman, N.G. , Proll, S. , Fredricks, D.N. et al. (2020) Complementing 16S rRNA gene amplicon sequencing with total bacterial load to infer absolute species concentrations in the vaginal microbiome. mSystems, 5(2). 10.1128/mSystems.00777-19 PMC7141891 32265316

[emi413216-bib-0038] Thoe, W. , Lee, O.H.K. , Leung, K.F. , Lee, T. , Ashbolt, N.J. , Yang, R.R. et al. (2018) Twenty five years of beach monitoring in Hong Kong: a re‐examination of the beach water quality classification scheme from a comparative and global perspective. Marine Pollution Bulletin, 131, 793–803. 29887007 10.1016/j.marpolbul.2018.05.002

[emi413216-bib-0039] USEPA . (2000) Improved enumeration methods for the recreational water quality indicators: *Enterococci* and *Escherichia coli*. *EPA‐821‐R‐97‐004* .

[emi413216-bib-0040] USEPA . (2003) Bacterial water quality standards for recreational waters. EPA‐823‐R‐03‐008.

[emi413216-bib-0041] Wang, Y.H. , Wu, Y.H. , Yu, T. , Zhao, X.H. , Tong, X. , Bai, Y. et al. (2019) Effects of chlorine disinfection on the membrane fouling potential of bacterial strains isolated from fouled reverse osmosis membranes. Science of the Total Environment, 693, 133579. 31376757 10.1016/j.scitotenv.2019.133579

[emi413216-bib-0042] Wanjugi, P. & Harwood, V.J. (2013) The influence of predation and competition on the survival of commensal and pathogenic fecal bacteria in aquatic habitats. Environmental Microbiology, 15, 517–526. 23013262 10.1111/j.1462-2920.2012.02877.x

[emi413216-bib-0043] Wear, S.L. , Acuña, V. , McDonald, R. & Font, C. (2021) Sewage pollution, declining ecosystem health, and cross‐sector collaboration. Biological Conservation, 255, 109010.

[emi413216-bib-0044] WHO . (1999) Health‐based monitoring of recreational waters: the feasibility of a new approach (the ‘Annapolis Protocol’). Geneva: World Health Organization (WHO).

[emi413216-bib-0045] Wong, Y.Y. , Lee, C.W. , Chai, S.C.Y. , Lim, J.H. , Bong, C.W. , Sim, E.U.H. et al. (2022) Distribution of faecal indicator bacteria in tropical waters of Peninsular Malaysia and their decay rates in tropical seawater. Marine Pollution Bulletin, 185, 114297. 36327936 10.1016/j.marpolbul.2022.114297

[emi413216-bib-0046] Xie, Y. , Liu, X. , Wei, H. , Chen, X. , Gong, N. , Ahmad, S. et al. (2022) Insight into impact of sewage discharge on microbial dynamics and pathogenicity in river ecosystem. Scientific Reports, 12(1), 6894. 35477966 10.1038/s41598-022-09579-x PMC9044725

[emi413216-bib-0047] Yoon, J.H. , Kang, K.H. , Oh, T.K. & Park, Y.H. (2004) Erythrobacter aquimaris sp. nov., isolated from sea water of a tidal flat of the Yellow Sea in Korea. International Journal of Systematic and Evolutionary Microbiology, 54, 1981–1985. 15545421 10.1099/ijs.0.63100-0

[emi413216-bib-0048] Zhang, Q. , He, X. & Yan, T. (2015) Differential decay of wastewater bacteria and change of microbial communities in beach sand and seawater microcosms. Environmental Science & Technology, 49, 8531–8540. 26125493 10.1021/acs.est.5b01879

