# Supplementary material for: Effects of chlorination on the survival of sewage bacteria in seawater microcosms
Source: Environ Microbiol Rep. 2023 Nov 21;16(1):e13216. doi: 10.1111/1758-2229.13216 (PMC10866060; doi:10.1111/1758-2229.13216)
Supplement: Supplementary file 1 — DATA S1: Supporting Information. [file EMI4-16-e13216-s001.pdf]

Supplementary materials

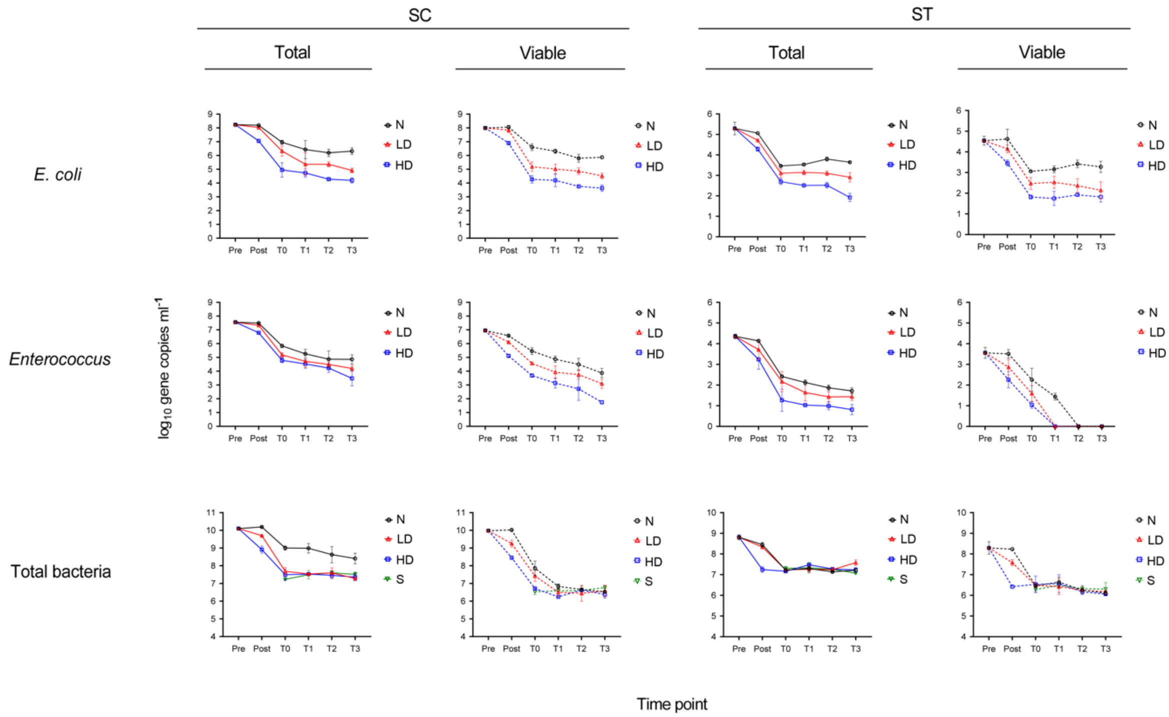

Figure S1. Total and viable concentrations of *E. coli*, *Enterococcus* and total bacteria in each sewage treatment work under different chlorine dosages and seawater control. The concentrations of total and viable cells in each group of bacteria were determined by qPCR assays and corrected with DNA extraction and PMA treatment efficiencies. Data are shown as mean ± 1 S.D. $\log_{10}$ copies per ml of effluent derived from three replicates. *E. coli* and *Enterococcus* were below detection in the seawater control. Pre and Post indicate samples before and after chlorination respectively. T0 indicates samples upon dilution with seawater, T1-T3 indicate samples taken from the microcosms at 24, 48 and 72 hr. N: no chorine; LD: low chlorine dosage; HD: high chlorine dosage; S: seawater only.

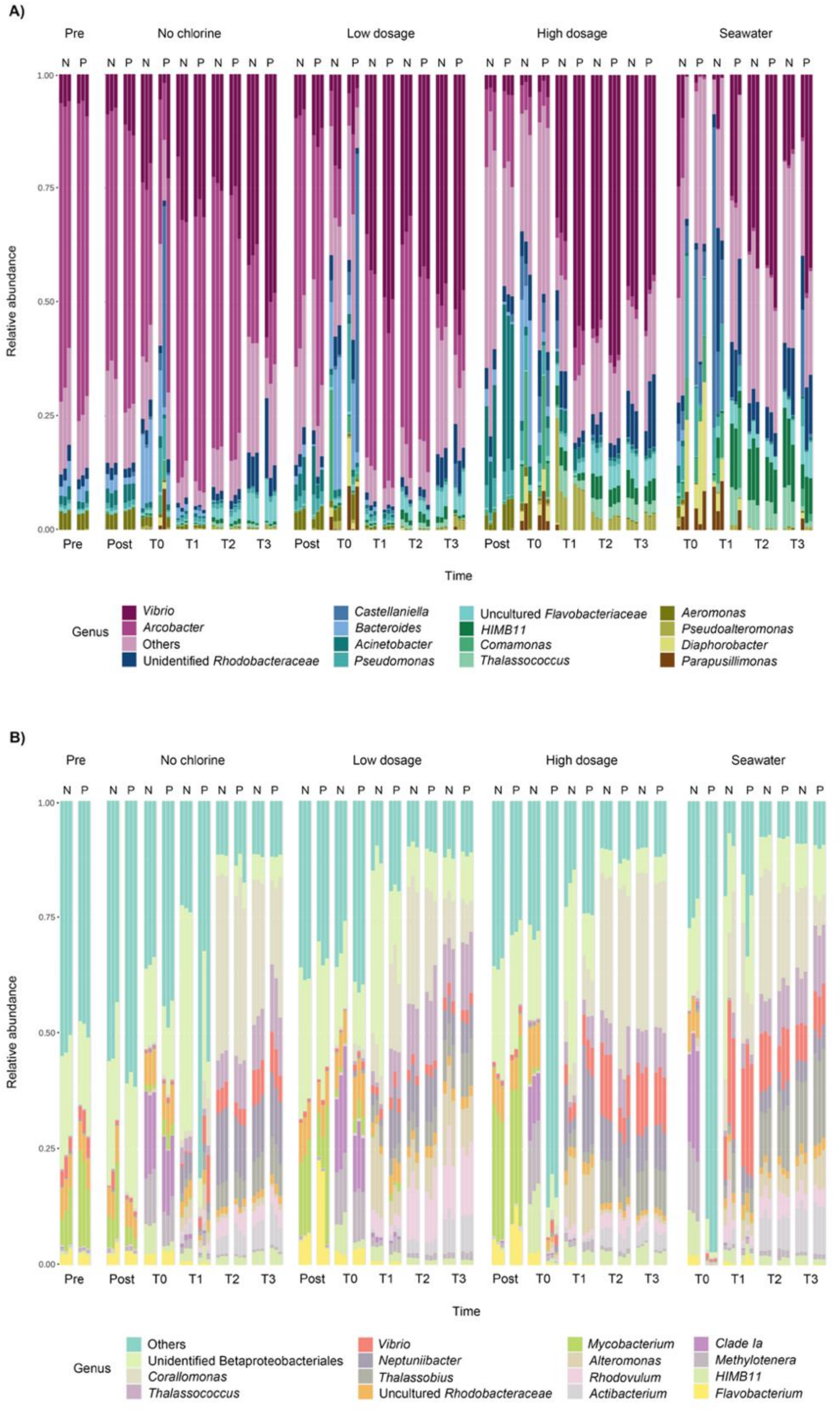

Figure S2. Top 15 abundant genera of the bacterial communities. Relative abundances of the abundant taxa in different treatment groups of samples from (A) SC and (B) ST are shown. Each bar represents individual triplicate. Pre and Post indicate samples before and after chlorination respectively. T0 indicates samples upon dilution with seawater, T1-T3 indicate samples taken from the microcosms at 24, 48 and 72 hr. N: no PMA treatment (total); P: with PMA treatment (viable).

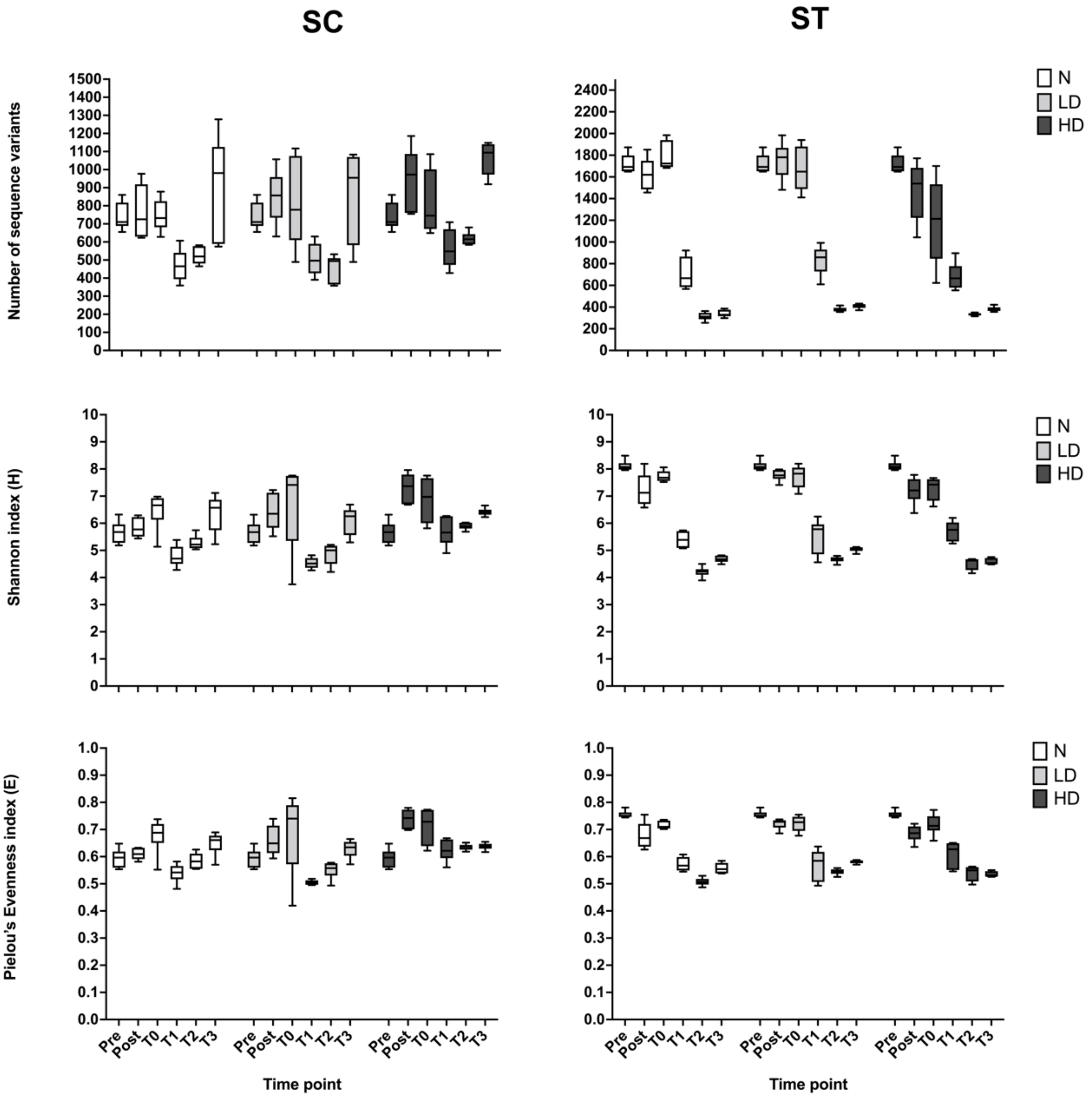

Figure S3. Alpha-diversity index boxplots (number of sequence variants, Shannon index and Pielou's evenness index) indicating the alterations in richness, diversity, and evenness of bacterial communities. All samples had been rarefied to the depth of 41169 reads. Pre and Post indicate samples before and after chlorination respectively. T0 indicates samples upon dilution with seawater, T1-T3 indicate samples taken from the microcosms at 24, 48 and 72 hr. N: no chorine; LD: low chlorine dosage; HD: high chlorine dosage.

Table S1. Physicochemical parameters of the samples in SC and ST. The total residual chlorine was measured at the end of chlorination in the low and high dosage sets. Measurements of all the physicochemical parameters were described in sections 2.2 and 2.4. Values are expressed as mean ± 1 S.D. Pre: effluents before chlorination; N: no chlorine; LD: low chlorine dosage; HD: high chlorine dosage; S: seawater control

Physicochemical parameters	Experimental set	SC	ST
Total residual chlorine (mg/L)	LD	0.1 ± 0.05	0.50 ± 0.03
	HD	1.03 ± 0.28	0.84 ± 0.07
Temperature (ºC)	Pre	29.4	25.7
	N	24.15 ± 1.41	22.49 ± 0.78
	LD	23.99 ± 1.08	24.27 ± 1.50
	HD	23.47 ± 2.25	22.67 ± 0.47
	S	24.1 ± 1.68	25.96 ± 1.85
Salinity (ppt)	Pre	12.02	0.16
	N	21.57 ± 6.14	26.57 ± 13.09
	LD	21.96 ± 6.44	26.65 ± 13.13
	HD	21.12 ± 6.16	26.89 ± 13.25
	S	32.28 ± 0.24	33.49 ± 0.38
Dissolved oxygen (mg/L)	Pre	1.90 ± 0.02	4.64
	N	4.67 ± 1.70	6.01 ± 0.68
	LD	4.81 ± 1.61	6.00 ± 1.10
	HD	5.19 ± 1.33	5.96 ± 0.99
	S	5.36 ± 0.16	5.26 ± 0.49
pH	Pre	7.08 ± 4.71e-3	6.66
	N	7.60 ± 0.36	7.60 ± 0.36
	LD	7.69 ± 0.38	7.68 ± 0.19
	HD	7.77 ± 0.40	7.70 ± 0.24
	S	7.95 ± 0.12	7.75 ± 0.10
Turbidity (NTU)	Pre	105.11 ± 0.24	51.76 ± 0.12
	N	7.63 ± 12.35	9.25 ± 16.15
	LD	11.36 ± 19.78	5.78 ± 9.31
	HD	13.21 ± 23.64	12.26 ± 22.21
	S	0.88 ± 0.27	1.89 ± 1.46
Biological oxygen demand (mg/L)	Pre	62.23 ± 0.12	6.13 ± 0.06
	N	19.89 ± 22.37	5.97 ± 0.65
	LD	18.93 ± 22.50	6.06 ± 0.71
	HD	19.57 ± 22.07	5.95 ± 0.83
	S	5.71 ± 3.45	5.44 ± 0.81
Total nitrogen (mg/L)	Pre	30.32 ± 0.88	1.46 ± 0.21
	N	6.43 ± 11.96	1.30 ± 1.57
	LD	6.58 ± 12.30	1.11 ± 1.49
	HD	6.86 ± 12.95	0.53 ± 0.06
	S	0.23 ± 0.07	0.50 ± 0.06
Ammonia (mg/L)	Pre	26.07 ± 0.32	0.23 ± 4.97e-3
	N	0.19 ± 0.21	0.01 ± 0.02
	LD	0.19 ± 0.21	0.01 ± 0.02
	HD	0.18 ± 0.21	0.01 ± 0.02
	S	0.01 ± 0.01	0.02 ± 0.03

Nitrite (mg/L)	Pre	0.03 ± 0.01	0.11 ± 3.92e-3
	N	0.01 ± 0.01	0.02 ± 0.01
	LD	0.01 ± 0.01	0.01 ± 4.96e-3
	HD	0.02 ± 0.01	0.01 ± 1.14e-3
	S	0.01	0.01 ± 6.26e-4
Nitrate (mg/L)	Pre	0.20 ± 0.09	0.63 ± 0.09
	N	0.18 ± 0.10	0.08 ± 0.11
	LD	0.24 ± 0.08	0.09 ± 0.15
	HD	0.22 ± 0.09	0.10 ± 0.17
	S	0.21 ± 0.08	0.01
Total phosphorous (mg/L)	Pre	2.13 ± 0.03	1.44 ± 0.02
	N	0.43 ± 0.77	0.31 ± 0.55
	LD	0.42 ± 0.76	0.32 ± 0.57
	HD	0.43 ± 0.76	0.31 ± 0.56
	S	0.02 ± 0.01	0.04 ± 0.03
Phosphate (mg/L)	Pre	1.27 ± 0.10	1.43 ± 0.02
	N	0.26 ± 0.49	0.26 ± 0.49
	LD	0.30 ± 0.57	0.29 ± 0.55
	HD	0.55 ± 0.29	0.29 ± 0.55
	S	0.01 ± 0.01	0.01 ± 0.01
Silicate (mg/L)	Pre	1.56 ± 4.93e-3	1.99 ± 0.02
	N	0.75 ± 0.56	0.78 ± 0.63
	LD	0.75 ± 0.50	0.77 ± 0.67
	HD	0.76 ± 0.48	0.74 ± 0.67
	S	0.30 ± 0.03	0.43 ± 0.06

Table S2. Primers and probes for qPCR assays. The target, gene, DNA sequences, amplicon size and references of each set of primers and probe are shown.

Target	Gene	Primer/probe	Sequence (5’-3’)	Amplicon size	Reference
Total bacteria	16S rRNA	Forward (331-F)	TCCTACGGGAGGCAGCAGT	466 bp	(1)
		Reverse (797-R)	GGACTACCAGGGTATCTAATCCTGTT		
		Probe	CGTATTACCGCGGCTGCTGGCAC		
Enterococcus	23S rRNA	Forward (ECST748F)	AGAAATTCCAAACGAACTTG	92 bp	(2)
		Reverse (ENC854R)	CAGTGCTCTACCTCCATCATT		
		Probe (GPL813TQ)	TGGTTCTCTCCGAAATAGCTTTAGGGCTA		
E. coli	23S rRNA	Forward	GGTAGAGCACTGTTTTGGCA	88 bp	(3)
		Reverse	TGTCTCCCGTGATAACTTTCTC		
		Probe	TCATCCCGACTTACCAACCCG		
Salmon testes (Sketa)	ITS region 2 of rRNA	Forward	GGTTTCCGCAGCTGGG	77 bp	(2)
		Reverse	CCGAGCCGTCCTGGTC		
		Probe	AGTCGCAGGCGGCCACCGT		
Universal DNA Spike-In Control	(Artificial sequence)	Forward	(Not provided by the company)	105 bp	(Thermo Fisher Scientific, Inc.)
		Reverse			
		Probe			

Reference

1. Nadkarni MA, Martin FE, Jacques NA, Hunter N. 2002. Determination of bacterial load by real-time PCR using a broad-range (universal) probe and primers set. Microbiology (N Y) 148:257–266.

2. USEPA. 2012. Method 1611: Enterococci in Water by TaqMan® Quantitative Polymerase Chain Reaction (qPCR) Assay. EPA-821-R-12-008.

3. Sivaganesan M, Aw TG, Briggs S, Dreelin E, Aslan A, Dorevitch S, Shrestha A, Isaacs N, Kinzelman J, Kleinheinz G, Noble R, Rediske R, Scull B, Rosenberg S, Weberman B, Sivy T, Southwell B, Siefring S, Oshima K, Haugland R. 2019. Standardized data quality acceptance criteria for a rapid Escherichia coli qPCR method (Draft Method C) for water quality monitoring at recreational beaches. Water Res 156:456–464.
